# Optimizing ward rounds: systematic review and meta-analysis of interventions to enhance patient safety

**DOI:** 10.1093/bjs/znaf041

**Published:** 2025-04-09

**Authors:** Ellie C Treloar, Jesse D Ey, Matheesha Herath, Nicholas P R Edwardes, Suzanne Edwards, Martin H Bruening, Guy J Maddern

**Affiliations:** Department of Surgery, The University of Adelaide, The Queen Elizabeth Hospital, Woodville, South Australia, Australia; Department of Surgery, The University of Adelaide, The Queen Elizabeth Hospital, Woodville, South Australia, Australia; Department of Surgery, The University of Adelaide, The Queen Elizabeth Hospital, Woodville, South Australia, Australia; Department of Surgery, The University of Adelaide, The Queen Elizabeth Hospital, Woodville, South Australia, Australia; School of Public Health, The University of Adelaide, Adelaide, South Australia, Australia; Department of Surgery, The University of Adelaide, The Queen Elizabeth Hospital, Woodville, South Australia, Australia; Department of Surgery, The University of Adelaide, The Queen Elizabeth Hospital, Woodville, South Australia, Australia

## Abstract

**Background:**

Poor quality ward rounds contribute to a large proportion of patient complications, delayed discharge, and increased hospital cost. This systematic review investigated all interventions aiming to improve patient and process-based outcomes in ward rounds.

**Methods:**

This systematic review was prospectively registered in PROSPERO, the international prospective register of systematic reviews (CRD42023394325). MEDLINE, Embase, Emcare, and PsycInfo were searched for studies with interventions aiming to improve ward round processes or patient outcomes in hospital settings. Studies were excluded if there was no baseline comparator or they were not in the ward round setting. Interventions were coded as checklist interventions (that is electronic or paper-based pro formas, templates, and checklists), structure interventions (that is defined rules or protocol to guide or standardize conduct), or other interventions. Outcomes were assessed via meta-analyses using the *I*^2^ statistic, Cochran’s Q *P* value, and random-effects models. Risk of bias was assessed using the Cochrane Risk of Bias 2 tool for RCTs and the Newcastle–Ottawa scale for non-randomized studies.

**Results:**

This review included 84 studies, from 18 countries, in 23 specialties, involving 43 570 patients. Checklist interventions significantly reduced ICU length of stay, improved overall documentation, and did not increase ward round duration. Structure interventions did not increase the time spent per patient or impact 30-day readmission rates or patient length of stay.

**Conclusion:**

This is the first systematic review with meta-analyses synthesizing the evidence of all ward round interventions targeted at improving patient and process outcomes. Results from this review should be used to inform guidelines for the ‘ideal ward round’.

## Teaser

This is the first systematic review with meta-analyses synthesizing the evidence of all ward round interventions targeted at improving patient and process outcomes. Results from this review should be used to inform guidelines or standards for the ‘ideal ward round’.

## Introduction

Every hospital admission involves a highly variable delivery of complex healthcare interventions, investigations, and procedures, simultaneously^1,2^. The multitude of moving parts necessary to care for each patient requires careful oversight and observation by the primary treating team. The ward round, in any specialty, serves as the primary checkpoint to ensure all aspects of a patient’s care are managed optimally^3,4^. Despite being a core component of clinical practice linked to patient outcomes, there is minimal literature informing or seeking to improve its practice^5–7^. Owing to the lack of clear guidelines, ward rounds are conducted primarily based on individual or departmental preference rather than evidence^3,4^. Time pressure, ineffective communication, poor documentation, and lack of standardization are key factors that contribute to poor quality ward rounds and preventable errors^8–12^.

A poor quality ward round can contribute to patient complications, delayed discharge, and subsequently increased hospital cost^9,13–15^. However, they are often overlooked as targets for process improvement^16–18^, with most interest focusing on the operating theatre and handover practice^4,13,19^. This is likely because of the variable nature of a ward round (numbers of staff, time obligations, structure, and patient complexities), which makes research in this area challenging. Furthermore, the lack of ward round standardization leads to poor identification and management of complications, resulting in significant variability of patient outcomes^5,13,19,20^. To date, there is a paucity of literature investigating the feasibility and efficacy of interventions to improve ward rounds. The aim of this review was to comprehensively assess all interventions that have been investigated to improve ward round outcomes (both patient and process related). These results can be utilized by governance bodies and working groups to inform best practice guidelines and directions for future research.

## Methods

### Search strategy and selection criteria

This review was registered in PROSPERO, the international prospective register of systematic reviews (CRD42023394325), and is reported in accordance with the PRISMA guidelines^21^. MEDLINE, Embase, Emcare, and PsycInfo were searched for studies published between January 1806 and 31 January 2023. Grey literature and conference proceedings were also examined and reference lists were pearled for additional relevant papers. The search terms were devised by a senior health reference librarian and a detailed search strategy is available in *Appendix S1*.

Screening was performed by two independent reviewers using the Covidence web-based systematic review management platform^21–23^. Conflicts were resolved by a third independent reviewer. Studies conducted in hospital ward round settings with any intervention (including, but not limited to, structure, time, simulation, coaching, interruptions, and checklists) aiming to improve ward round processes, ward round quality, or patient outcomes were included. The outcomes included process-based outcomes (including, but not limited to, documentation rates, interruptions, cost, and time taken per patient) and patient outcomes (including, but not limited to, patient cognitive and psychological outcomes, length of stay, and complications). Observational and experimental studies in all medical and surgical specialties, in adult or paediatric settings, and published in full were included. Where studies were not published in English, Google translate was utilized for screening. Studies not in the ward round setting or those that did not have a baseline group or involve the primary medical team were excluded. Studies that measured individuals’ subjective experiential outcomes (perception, burnout, and fatigue) as main outcomes or studies with interventions comprising the addition of non-medical personnel, such as allied health personnel, were also excluded.

### Data analysis

**Fig. 1:**
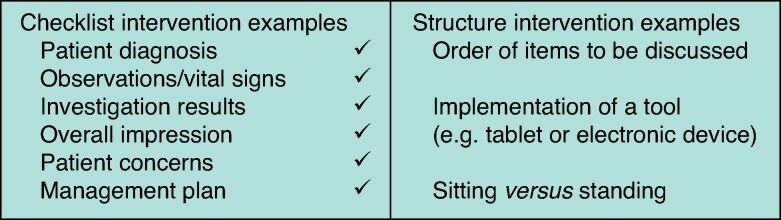
Examples of checklist and structure interventions in ward rounds

Data extraction was performed by two independent authors using a pre-formulated data extraction plan (*Appendix S2*). Intervention types were coded and outcomes within intervention types were assessed via meta-analysis (if appropriate results were available) or qualitative analysis. Interventions were coded as checklist interventions, structure interventions, or other interventions. Checklist interventions could be paper based or electronic, and included pro formas, checklists, and templates. Structure interventions included any interventions aiming to change how the ward round was conducted or provide standardization. Examples of this would be protocolized processes with specific steps to be followed, sitting *versus* standing rounds, and assigning roles to team members. See *Figure 1* for examples of checklist and structure interventions in ward rounds.

Data analyses were performed using Stata Statistical Software: Release 15.1 (StataCorp LP, College Station, TX, USA). The *I*^2^ statistic was used to evaluate heterogeneity (with *I*^2^ >50% indicating significant heterogeneity) as was Cochran’s Q *P* value (with a *P* value <0.100 indicating significant heterogeneity). Random-effects models were used throughout. A *P* value of ≤0.050 denoted statistical significance. A variable was included in a forest plot if at least two of the journal articles involved had sufficient values for that variable. Outcome variables for each intervention group and control were assessed using either the standardized mean difference (MD) or the risk ratio (RR) and the 95% confidence interval. All outcomes were combined in forest plots, displaying an MD or RR and 95% c.i. for the outcomes for checklist interventions and the outcomes for structure interventions.

Risk of bias was assessed independently by two reviewers using the Cochrane Risk of Bias 2 (RoB 2) tool for RCTs and the Newcastle–Ottawa Scale for non-randomized studies^24,25^. Discrepancies were resolved by discussion.

## Results

### Search results

**Fig. 2:**
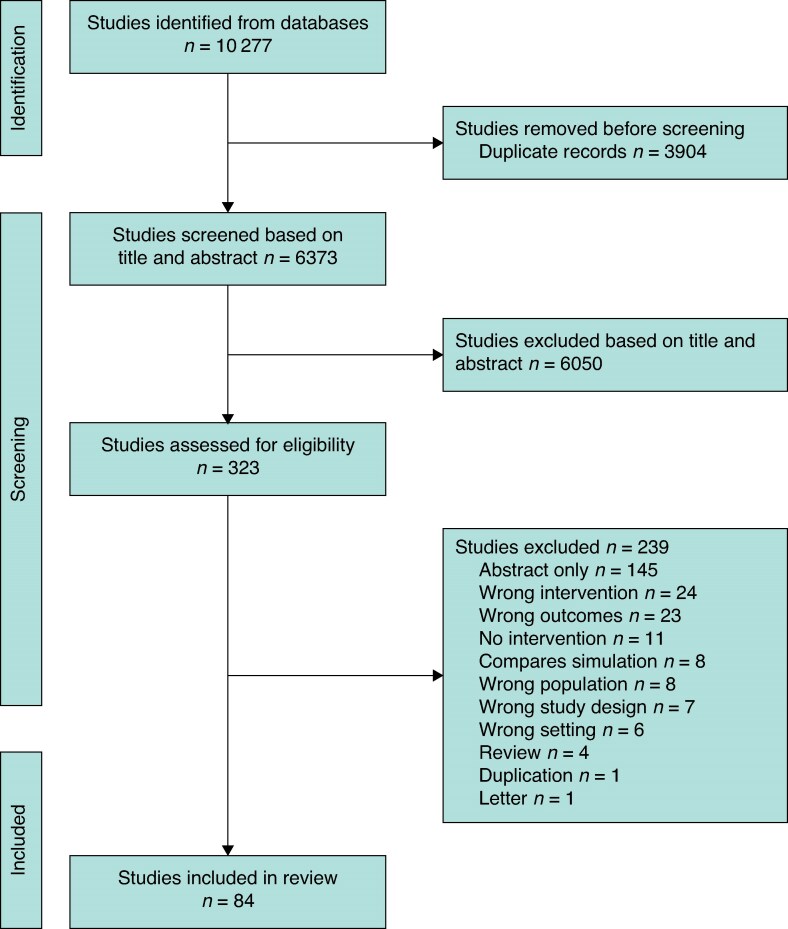
PRISMA flow diagram

The preliminary search yielded 10 277 articles. After removal of duplicates, title and abstract screening, and full-text screening, 84 studies were included (*Fig. 2*). A table of all included studies is available in *Appendix S3* and a table of excluded studies and the reason for exclusion is available in *Appendix S4*.

### Study characteristics

The 84 studies, involving 43 570 patients, consisted of 72 cohort studies^9,26–96^ and 12 RCTs^97–108^. Checklist interventions were the most common intervention type (51 studies), followed by structure interventions (24 studies) and other interventions (9 studies); other interventions included staff education (5 studies), timing (1 study), a patient information sheet (1 study), a traffic light system (1 study), and application of athletic principles (1 study).

There was significant heterogeneity in outcome measures and results reported across the 84 publications. Many studies gathered data on multiple endpoints, with the most common being documentation (37 studies), followed by ward round duration (15 studies), patient satisfaction (12 studies), and hospital length of stay (9 studies); however, the methodology for reporting patient satisfaction varied significantly.

### Checklist interventions

#### Meta-analysis

**Fig. 3:**
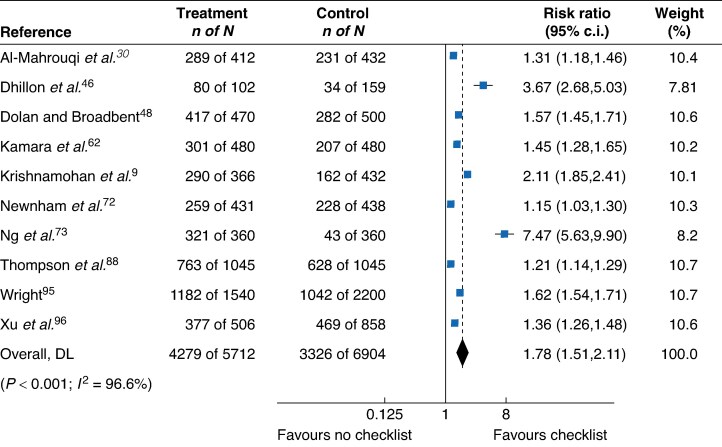
Overall documentation meta-analysis Forest plot comparing all studies reporting the impact of a checklist intervention on overall documentation. Weights are from a random-effects model. DL, Overall effect size calculated using DerSimonian-Laird (DL) method.

**Fig. 4:**
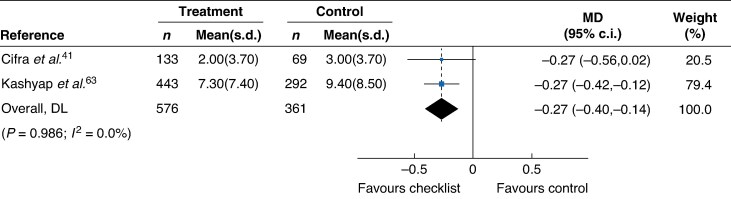
ICU length of stay meta-analysis Forest plot comparing all studies reporting the impact of a checklist intervention on ICU length of stay. Weights are from a random-effects model. MD, mean difference; DL, Overall effect size calculated using DerSimonian-Laird (DL) method.

Standardized comparisons of overall documentation, ICU length of stay, hospital length of stay, ICU mortality, and inpatient mortality before and after interventions were conducted by pooling the studies using random-effects models. It was found that checklist interventions increased overall documentation compared with no checklist intervention (RR 1.78 (95% c.i. 1.51 to 2.11)) (*Fig. 3*). Checklist interventions were also found to significantly reduce ICU length of stay compared with control (MD −0.27 (95% c.i. −0.40 to −0.14)) (*Fig. 4*). However, checklist interventions had no significant impact on hospital length of stay (MD −0.104 (95% c.i. −2.60 to 0.51)) (*Appendix S5*), ICU mortality (RR 0.84 (95% c.i. 0.59 to 1.19)) (*Appendix S6*), and inpatient mortality (RR 0.79 (95% c.i. 0.57 to 1.09)) (*Appendix S7*).

We performed 25 individual meta-analyses that compared specific documentation rates before and after exposure to the checklist intervention. These meta-analyses demonstrated that a checklist intervention significantly improved documentation of the following 12 specific points: observations (RR 1.68 (95% c.i. 1.01 to 2.78)), diagnosis (RR 1.53 (95% c.i. 1.08 to 2.17)), impression (RR 2.13 (95% c.i. 1.36 to 3.34)), deep-vein thrombosis (RR 6.54 (95% c.i. 3.56 to 12.03)), resuscitation status (RR 6.89 (95% c.i. 1.42 to 33.47)), drug chart review (RR 2.69 (95% c.i. 1.63 to 4.43)), bloods (RR 1.95 (95% c.i. 1.29 to 2.94)), venous thromboembolism (VTE) (RR 4.63 (95% c.i. 2.32 to 9.24)), bleep (RR 1.2 (95% c.i. 1.06 to 1.35)), ECG (RR 1.4 (95% c.i. 1.02 to 1.92)), plan (RR 1.11 (95% c.i. 1.02 to 1.21)), and discharge planning (RR 3.19 (95% c.i. 1.41 to 7.22)). Meta-analysis forest plots are available in *Appendix S8*.

The meta-analyses demonstrated that a checklist intervention did not significantly improve documentation of the following 12 points: lead of the ward round, time, signature, date, dietary plan, hospital number, examination, consultant name, chest X-ray (CXR), grade, patient name, and vitals. Meta-analysis forest plots are available in *Appendix S9*.

#### Qualitative summary

There were 51 studies investigating checklist interventions, including 10 pro formas and 41 checklists; 43 were paper based and 8 were electronic. The number of checklist items ranged between 6 and 29, with a median of 13. There were 169 different items on the checklists (38 items appeared more than 5 times). Frequency analyses revealed that the ten most common checklist items were diagnosis, observations, impression, bloods, VTE, examination findings, patient concerns, antibiotic review, dietary plan, and plan. There were no statistically significant associations between checklist interventions and either ward round duration or hospital readmission rates in any study^26,35,41,42,50,77,79,87,90,91^. Several studies investigated checklist interventions and other patient outcomes (urinary tract infection rates, infection rates, duration of intravenous fluids and lines, patient satisfaction, antibiotic use, and VTE status). *Appendix S10* synthesizes the checklist interventions and qualitative outcomes.

### Structure interventions

#### Meta-analysis

Meta-analyses indicated that ward round structure interventions did not significantly increase the time spent rounding per patient (MD −0.74 (95% c.i. −55.46 to 53.97)) (*Appendix S11*) or in total (MD 1364.14 (95% c.i. −407.52 to 3135.79)) (*Appendix S12*) but did not have a significant impact on 30-day readmission (MD 0.93 (95% c.i. 0.63 to 1.36)) (*Appendix S13*) or patient length of stay (MD −0.15 (95% c.i. −0.31 to 0.01)) (*Appendix S14*).

#### Qualitative summary

Associations between ward round structure interventions and patient-centred outcomes, such as rate of falls, postoperative morbidity, length of stay, and discharge time, were investigated in several studies. *Appendix S15* synthesizes these outcomes.

### Other interventions

Associations between other interventions and patient-centred outcomes were investigated in nine studies, but these were not included in meta-analyses. Education interventions were associated with improved patient satisfaction, documentation of goals of care, and change in management; however, no statistical analysis was reported^84,105,109^. An education intervention did significantly improve shared decision-making in one study^59^. The novel traffic light system was suggested to improve bed turnover rate^89^, delayed rounds (*versus* early) were linked to higher patient satisfaction^107^, and a patient information sheet significantly improved a patient's knowledge regarding their treatment plan for the day^70^. *Appendix S16* synthesizes the other interventions and qualitative outcomes.

#### Bias assessment

Included observational studies scored between two and seven out of a possible nine stars using the Newcastle–Ottawa scale (see *Appendix S17*). Of the included RCTs, using the Cochrane RoB 2 tool, 4 of 12 studies were high risk, 2 of 12 studies had some concerns, and 6 of 12 were low risk (see *Appendix S18*).

## Discussion

This systematic review provides a comprehensive analysis of all interventions previously investigated to improve patient and process-based outcomes in ward rounds globally. Results from the 84 included studies and 34 meta-analyses suggest that checklist interventions and structure interventions can improve patient care in the ward round. Checklist interventions were demonstrated to reduce length of stay in the ICU and improve overall and specific documentation of discussion on the round, whilst not increasing ward round duration per patient. No study that demonstrated a reduced length of stay in the ICU continued to use a checklist throughout the rest of the patient’s hospitalization after ICU stay. This presents an opportunity for further exploration of longitudinal care using a checklist for the entirety of a patient’s admission.

This systematic review not only includes the first meta-analysis demonstrating that checklist interventions improve overall documentation in ward rounds, but also demonstrates in a further 25 meta-analyses that a checklist improved 12 of 25 specific points on the checklist. However, checklist interventions did not significantly affect hospital length of stay, ICU mortality, or inpatient mortality. Although checklist interventions did not result in significant improvements in hospital length of stay, ICU mortality, or inpatient mortality, they did result in several other significant patient and process-based outcomes. These improvements included improved documentation rates improved prescribing, improved patient satisfaction, reduced infections, and reduced adverse events (described in the qualitative table). Checklist interventions are simple to introduce and relatively inexpensive. Considering no reported downside and several significant improvements, they are a good starting point for improving different aspects of care in the surgical ward round. However, to achieve more robust improvements, in terms of reducing length of stay and mortality, other interventions may be necessary.

The meta-analyses on structure interventions indicated that a change of structure did not increase the time spent rounding per patient and did not impact patient outcomes. Given hesitation to adopt new methodologies due to time constraints^110^, it is important that no significant time difference was observed. Multiple other interventions were trialled, but, owing to the vast heterogeneity regarding design and outcomes, and lack of statistical rigour, are unable to guide evidence-based practice.

A multitude of intervention types have been investigated with various objectives and endpoints. This is important because the ward round underpins the quality of daily care patients are receiving and dictates hospital bed availability^17^. Concerningly, only 12 studies assessed patient satisfaction. Higher patient satisfaction is known to increase compliance and adherence to treatment, involvement in care, and positive health-related behaviour^111,112^. Future studies assessing new ward round interventions should therefore ensure inclusion of patient perspectives and satisfaction regarding interventions.

The highly variable nature of ward rounds (regarding design, procedure, and time pressures) fosters an environment where omissions and errors can be made^4^. Preventable adverse errors may often occur on the ward round, yet there is still no ‘gold standard’ or consensus for the ‘ideal ward round’^15,113,114^. Recommendations based on current literature in 2012^7^ saw subsequent studies focusing on improving components of the ward round, which resulted in the second update of the guidance (2021) and National Institute for Health and Care Excellence (NICE) guidelines for structuring ward rounds using checklists^17,115^. Future research may need to be more granular to first identify the specific needs and issues that affect individual institutions, specialties, and wards before interventions are implemented. This could be done through review of documentation/records to identify the point at which the hospital process became suboptimal and what prevention strategies need to be adopted^15,116,117^.

A limitation of this study is that a significant number of the included studies were observational. These were important to include as they represented the majority of research on this subject, yet they are highly biased. Next, the substantial heterogeneity of specific intervention components within each intervention code type limits generalizability in terms of implementation. These results demonstrate that checklists are effective interventions to improve aspects of ward rounds, but specifics pertaining to the number and content of checklist items and the modality of delivery (electronic *versus* paper based) to provide the best results are, as yet, unknown. Future studies should utilize the positive findings from this systematic review to focus attention on areas that warrant further high-quality studies.

The delivery of high-quality ward rounds is challenging^17^. A bottom-up and patient-focused approach is needed to improve the ward round, combining evidence, clinical practice, and key stakeholders^118,119^. Interventions must not add to the burden of clinicians, but should improve and foster teamwork within groups, be patient focused, and improve patient satisfaction^118^.

It is crucial to determine how to ‘best adapt the traditional ward-round process to suit a continually evolving, complex system’^7^. This systematic review is the first step in providing a thorough evidence base regarding which interventions can improve process and patient-based ward round outcomes and should be used in collaboration with governance bodies, quality improvement teams, and working groups (doctors, nursing staff, and allied health personnel) to inform new guidelines and best practice.

## Supplementary Material

znaf041_Supplementary_Data

## Data Availability

Additional data can be made available upon reasonable request.

## References

[1] Johnson H, O'Farrell A, McKeown D, Sayers G, Hayes C, Beaton D. Is increasing life expectancy leading to more complexity? Ir Med J 2018;111:672 29869853

[2] Committee on the Learning Health Care System in America—Institute of Medicine . Best Care at Lower Cost: The Path to Continuously Learning Health Care in America. Washington, DC: The National Academies Press, 2013 24901184

[3] Nikendei C, Kraus B, Schrauth M, Briem S, Junger J. Ward rounds: how prepared are future doctors? Med Teach 2008;30:88–91 18278658 10.1080/01421590701753468

[4] Cohn A . The ward round: what it is and what it can be. Br J Hosp Med (Lond) 2014; 75(Suppl 6): C82–C85 25075416

[5] Pucher PH, Aggarwal R, Srisatkunam T, Darzi A. Validation of the simulated ward environment for assessment of ward-based surgical care. Ann Surg 2014;259:215–221 23470580 10.1097/SLA.0b013e318288e1d4

[6] Ghaferi AA, Birkmeyer JD, Dimick JB. Variation in hospital mortality associated with inpatient surgery. N Engl J Med 2009;361:1368–1375 19797283 10.1056/NEJMsa0903048

[7] Royal College of Physicians, Royal College of Nursing. Ward Rounds in Medicine: Principles for Best Practice. London: Royal College of Physicians, 2012

[8] General Medical Council . Good Medical Practice. Manchester: General Medical Council, 2013

[9] Krishnamohan N, Maitra I, Shetty VD. The surgical ward round checklist: improving patient safety and clinical documentation. J Multidiscip Healthc 2019;12:789–794 31571896 10.2147/JMDH.S178896 PMC6754526

[10] Zegers M, de Bruijne MC, Spreeuwenberg P, Wagner C, Groenewegen PP, van der Wal G. Quality of patient record keeping: an indicator of the quality of care? BMJ Qual Saf 2011;20:314–318 10.1136/bmjqs.2009.038976 21303769

[11] Shetty K, Poo SXW, Sriskandarajah K, Sideris M, Malietzis G, Darzi A et al “The longest way round is the shortest way home”: an overhaul of surgical ward rounds. World J Surg 2018;42:937–949 29067515 10.1007/s00268-017-4267-1 PMC5843677

[12] Wilson RM, Runciman WB, Gibberd RW, Harrison BT, Newby L, Hamilton JD. The quality in Australian health care study. Med J Aust 1995;163:458–471 7476634 10.5694/j.1326-5377.1995.tb124691.x

[13] Pucher PH, Aggarwal R, Darzi A. Surgical ward round quality and impact on variable patient outcomes. Ann Surg 2014;259:222–226 24263319 10.1097/SLA.0000000000000376

[14] Fernando KJ, Siriwardena AK. Standards of documentation of the surgeon-patient consultation in current surgical practice. Br J Surg 2001;88:309–312 11167887 10.1046/j.1365-2168.2001.01666.x

[15] Neale G, Woloshynowych M, Vincent C. Exploring the causes of adverse events in NHS hospital practice. J R Soc Med 2001;94:322–330 11418700 10.1177/014107680109400702 PMC1281594

[16] Pucher PH, Aggarwal R. Re: Does surgical ward round quality really impact on patient outcomes? AnnSurg 2016;263:e10 10.1097/SLA.0000000000001023 25371127

[17] Royal College of Physicians, Royal College of Nursing. Modern Ward Rounds: Good Practice for Multidisciplinary Inpatient Review. London: Royal College of Physicians, 2021

[18] O'Hare JA . Anatomy of the ward round. Eur J Intern Med 2008;19:309–313 18549930 10.1016/j.ejim.2007.09.016

[19] Klingensmith ME . Ward rounds and patient outcome: be attentive or suffer the peril. Ann Surg 2014;259:227–228 24398924 10.1097/SLA.0000000000000493

[20] Ghaferi AA, Birkmeyer JD, Dimick JB. Hospital volume and failure to rescue with high-risk surgery. Med Care 2011;49:1076–1081 22002649 10.1097/MLR.0b013e3182329b97

[21] Page MJ, McKenzie JE, Bossuyt PM, Boutron I, Hoffmann TC, Mulrow CD et al The PRISMA 2020 statement: an updated guideline for reporting systematic reviews. BMJ 2021;372:n71 33782057 10.1136/bmj.n71 PMC8005924

[22] Moher D, Liberati A, Tetzlaff J, Altman DG. Preferred reporting items for systematic reviews and meta-analyses: the PRISMA statement. Ann Intern Med 2009;151:264–269 19622511 10.7326/0003-4819-151-4-200908180-00135

[23] Kellermeyer L, Harnke B, Knight S. Covidence and Rayyan. J Med Libr Assoc 2018;106:580–583

[24] Luchini C, Stubbs B, Solmi M, Veronese N. Assessing the quality of studies in meta-analyses: advantages and limitations of the Newcastle Ottawa scale. World J Meta-Anal 2017;5:80–84

[25] Minozzi S, Cinquini M, Gianola S, Gonzalez-Lorenzo M, Banzi R. The revised Cochrane risk of bias tool for randomized trials (RoB 2) showed low interrater reliability and challenges in its application. J Clinepidemiol 2020;126:37–44 10.1016/j.jclinepi.2020.06.015 32562833

[26] Abraham J, Jaros J, Ihianle I, Kochendorfer K, Kannampallil T. Impact of EHR-based rounding tools on interactive communication: a prospective observational study. Int J Med Inform 2019;129:423–429 31445286 10.1016/j.ijmedinf.2019.07.012

[27] Acal Jimenez R, Swartz M, McCorkle R. Improving quality through nursing participation at bedside rounds in a pediatric acute care unit: a pilot project. J Pediatr Nurs 2018;43:45–55 30473156 10.1016/j.pedn.2018.08.010

[28] Banfield DA, Adamson C, Tomsett A, Povey J, Fordham T, Richards SK. ‘Take Ten’ improving the surgical post-take ward round: a quality improvement project. BMJ Open Qual 2018;7:e000045 10.1136/bmjoq-2017-000045 PMC5841505 29527575

[29] Alazzawi S, Silk Z, Saha UU, Auplish S, Masterson S. A ward round proforma improves documentation and communication. Br J Hosp Med 2016;77:712–716 10.12968/hmed.2016.77.12.712 27937015

[30] Al-Mahrouqi H, Oumer R, Tapper R, Roberts R. Post-acute surgical ward round proforma improves documentation. BMJ Qual Improv Rep 2013;2:u201042.w688 10.1136/bmjquality.u201042.w688 PMC4652723 26734192

[31] Armstrong EJ, Carpenter KJ. A standardized ward round proforma improves documentation in a specialist stroke unit. Cureus 2022;14:e31931 36447809 10.7759/cureus.31931 PMC9701495

[32] Blucher KM, Dal Pra SE, Hogan J, Wysocki AP. Ward safety checklist in the acute surgical unit. ANZ J Surg 2014;84:745–747 24341940 10.1111/ans.12496

[33] Boland X . Implementation of a ward round pro-forma to improve adherence to best practice guidelines. BMJ Qual Improv Rep 2015;4:u207456.w2979 10.1136/bmjquality.u207456.w2979 PMC4645828 26734332

[34] Brown N, Horne J, Low A. Improving documentation and junior doctor confidence on COVID-19 ward rounds using a ward round pro forma. Clin Med (Lond) 2021; 21(Suppl 2): 17–18 34078680 10.7861/clinmed.21-2-s17

[35] Brown OS, Toi TH, Barbosa PR, Pookarnjanamorakot P, Trompete A. A patient-centred check sheet improves communication on the trauma ward round. Br J Hosp Med (Lond) 2019;80:472–475 31437033 10.12968/hmed.2019.80.8.472

[36] Byrd AS, McMahon PM, Vath RJ, Bolton M, Roy M. Integration of mobile devices to facilitate patient care and teaching during family-centered rounds. Hosp Pediatr 2018;8:44–48 29217525 10.1542/hpeds.2016-0193

[37] Cao V, Horn F, Laren T, Scott L, Giri P, Hidalgo D et al Patient-centered structured interdisciplinary bedside rounds in the medical ICU. Crit Care Med 2016; 44(Suppl 1): 346 10.1097/CCM.0000000000002807 29088002

[38] Chow MY, Nikolic S, Shetty A, Lai K. Structured interdisciplinary bedside rounds in an Australian tertiary hospital emergency department: patient satisfaction and staff perspectives. Emerg Med Australas 2019;31:347–354 30126054 10.1111/1742-6723.13160

[39] Christensen K, Janssens S, Beckmann M. Evaluation of a standardized ward round in a prenatal inpatient setting. Int J Gynaecol Obstet 2017;136:357–361 28087887 10.1002/ijgo.12080

[40] Christianson K, Kalinowski A, Bauer S, Liu Y, Titus L, Havas M et al Using quality improvement methodology to increase communication of discharge criteria on rounds. Hosp Pediatr 2022;12:156–164 34988584 10.1542/hpeds.2021-006127

[41] Cifra CL, Houston M, Otto A, Kamath SS. Prompting rounding teams to address a daily best practice checklist in a pediatric intensive care unit. Jt Comm J Qual Patient Saf 2019;45:543–551 31326347 10.1016/j.jcjq.2019.05.012

[42] Clark NA, Burrus S, Richardson T, Sterner S, Queen MA. Implementation of a general pediatric clinical rounding checklist. Hosp Pediatr 2019;9:291–299 30902823 10.1542/hpeds.2018-0150

[43] Conroy KM, Elliott D, Burrell AR. Testing the implementation of an electronic process-of-care checklist for use during morning medical rounds in a tertiary intensive care unit: a prospective before-after study. Ann Intensive Care 2015;5:60 26239145 10.1186/s13613-015-0060-1 PMC4523566

[44] Crowson MG, Kahmke R, Ryan M, Scher R. Utility of daily mobile tablet use for residents on an Otolaryngology Head & Neck Surgery inpatient service. J Med Sys 2016;40:55 10.1007/s10916-015-0419-8 26645319

[45] De Bie AJR, Mestrom E, Compagner W, Nan S, van Genugten L, Dellimore K et al Intelligent checklists improve checklist compliance in the intensive care unit: a prospective before-and-after mixed-method study. Br J Anaesth 2021;126:404–414 33213832 10.1016/j.bja.2020.09.044

[46] Dhillon P, Murphy RKJ, Ali H, Burukan Z, Corrigan MA, Sheikh A et al Development of an adhesive surgical ward round checklist; a technique to improve patient safety. Ir Med J 2011;104:303–305 22256442

[47] Dodek PM, Norena M, Wong H, Keenan S, Martin C. Assessing the influence of intensive care unit organizational factors on outcomes in Canada: is there residual confounding? J Intensive Care Ced 2015;30:413–419 10.1177/0885066614521973 24509494

[48] Dolan R, Broadbent P. A quality improvement project using a problem based post take ward round proforma based on the SOAP acronym to improve documentation in acute surgical receiving. Ann Med Surg (Lond) 2015;5:45–48 26858834 10.1016/j.amsu.2015.11.011 PMC4706565

[49] Duxbury O, Hili S, Afolayan J. Using a proforma to improve standards of documentation of an orthopaedic post-take ward round. BMJ Qual Improv Rep 2013;2:u200902.w699 10.1136/bmjquality.u200902.w699 PMC4652710 26734180

[50] Eden EL, Rothenberger S, DeKosky A, Donovan AK. The safe discharge checklist: a standardized discharge planning curriculum for medicine trainees. South Med J 2022;115:18–21 34964055 10.14423/SMJ.0000000000001341

[51] Efune PN, Morse RB, Sheehan M, Malone LM, Robertson TS, Darnell C. Improving reliability to a care goal rounding template in the pediatric intensive care unit. Pediatr Qual Saf 2018;3:e117 31334449 10.1097/pq9.0000000000000117 PMC6581481

[52] Escamilla-Ocanas CE, Torrealba-Acosta G, Mandava P, Qasim MS, Gutierrez-Flores B, Bershad E et al Implementation of systematic safety checklists in a neurocritical care unit: a quality improvement study. BMJ Open Qual 2022;11:e001824 10.1136/bmjoq-2022-001824 PMC9743379 36588320

[53] Feinman M, Hsu ATW, Taylor S, Torbeck L. Cutting the fat: utilizing LEAN methodology to improve rounding efficiency of surgical residents. Am J Surg 2022;223:1100–1104 34916037 10.1016/j.amjsurg.2021.12.005

[54] Fleischmann R, Duhm J, Hupperts H, Brandt SA. Tablet computers with mobile electronic medical records enhance clinical routine and promote bedside time: a controlled prospective crossover study. J Neurol 2015;262:532–540 25476692 10.1007/s00415-014-7581-7 PMC4363516

[55] Galloway GK, Choudhury SN. New take on the post-take ward round: a quality improvement project undertaken in a district general hospital. BMJ Open Qual 2022;11:e001923 10.1136/bmjoq-2022-001923 PMC9535189 36192036

[56] Gilliland N, Catherwood N, Chen S, Browne P, Wilson J, Burden H. Ward round template: enhancing patient safety on ward rounds. BMJ Open Qual 2018;7:e000170 10.1136/bmjoq-2017-000170 PMC5926569 29719873

[57] Glick AF, Foster LZ, Goonan M, Hart LH, Alam S, Rosenberg RE. Using quality improvement science to promote reliable communication during family-centered rounds. Pediatr 2022;149:e2021050197 10.1542/peds.2021-050197 PMC9647567 35362064

[58] Hale G, McNab D. Developing a ward round checklist to improve patient safety. BMJ Qual Improv Rep 2015;4:u204775.w2440 10.1136/bmjquality.u204775.w2440 PMC4645926 26734369

[59] Harman SM, Blankenburg R, Satterfield JM, Monash B, Rennke S, Yuan P et al Promoting shared decision-making behaviors during inpatient rounds: a multimodal educational intervention. Acad Med 2019;94:1010–1018 30893066 10.1097/ACM.0000000000002715 PMC6594883

[60] Johnston J, Stephenson J, Rajgopal A, Bhasin N. ‘Every patient, every day’: a daily ward round tool to improve patient safety and experience. BMJ Open Qual 2022;11:e001829 10.1136/bmjoq-2022-001829 PMC9528666 36171004

[61] Justice LB, Cooper DS, Henderson C, Brown J, Simon K, Clark L et al Improving communication during cardiac ICU multidisciplinary rounds through visual display of patient daily goals. Pediatr Crit Care Med 2016;17:677–683 27176731 10.1097/PCC.0000000000000790

[62] Kamara A, Henderson S, Rodrigo C, Dulay J. Does a post-take ward round proforma lead to sustainable improvements in quality of documentation for patients admitted to the medical assessment unit? Acute Med 2006;5:108–111 21611627

[63] Kashyap R, Murthy S, Arteaga GM, Dong Y, Cooper L, Kovacevic T et al Effectiveness of a daily rounding checklist on processes of care and outcomes in diverse pediatric intensive care units across the world. J Trop Pediatr 2021;67:fmaa058 32853362 10.1093/tropej/fmaa058 PMC8488874

[64] Keller C, Arsenault S, Lamothe M, Bostan SR, O'Donnell R, Harbison J et al Patient safety ward round checklist via an electronic app: implications for harm prevention. Ir J Med Sci 2018;187:553–559 29110186 10.1007/s11845-017-1687-8

[65] Khan A, Spector ND, Baird JD, Ashland M, Starmer AJ, Rosenbluth G et al Patient safety after implementation of a coproduced family centered communication programme: multicenter before and after intervention study. BMJ 2018;363:k4764 30518517 10.1136/bmj.k4764 PMC6278585

[66] Koumoullis HD, Shapev M, Wong G, Gerring S, Patrinios G, Depasquale I et al Improving the quality of the daily ward round in a plastic surgery unit by adapting the SAFE ward round tool of the Royal College of Surgeons of Edinburgh. J Patient Saf Risk Manag 2020;25:233–238

[67] Lepee C, Klaber RE, Benn J, Fletcher PJ, Cortoos P-J, Jacklin A et al The use of a consultant-led ward round checklist to improve paediatric prescribing: an interrupted time series study. Eur J Pediatr 2012;171:1239–1245 22628136 10.1007/s00431-012-1751-3

[68] Licata J, Aneja R, Pasek T, Kyper C, Miller E, Spencer T et al A foundation for patient safety: phase I implementation of interdisciplinary bedside rounds in the pediatric intensive care unit. Criti Care Med 2011; 39(Suppl 12): 172 10.4037/ccn2013280 23727856

[69] Ludley A, Bahk A, Al-Shihabi A. A structured rounding proforma in the hyper acute stroke unit (HASU): a quality improvement project. J Health Qual 2023;45:10–18 10.1097/JHQ.0000000000000364 36584114

[70] Murphy D, Crowley R, Spencer A, Birch M. When can I go home? A prospective case control study to improve communication with patients regarding their diagnosis, treatment plan and likely discharge date. N Z Med J 2015;128:53–58 25899493

[71] Nassikas NJ, Monteiro JFG, Pashnik B, Lynch J, Carino G, Levinson AT. Intensive care unit rounding checklists to reduce catheter-associated urinary tract infections. Infect Control Hosp Epidemiol 2020;41:680–683 32127059 10.1017/ice.2020.43

[72] Newnham AL, Hine C, Rogers C, Agwu JC. Improving the quality of documentation of paediatric post-take ward rounds: the impact of an acrostic. Postgrad Med J 2015;91:22–25 25476019 10.1136/postgradmedj-2013-132534

[73] Ng J, Abdelhadi A, Waterland P, Swallow J, Nicol D, Pandey S et al Do ward round stickers improve surgical ward round? A quality improvement project in a high-volume general surgery department. BMJ Open Qual 2018;7:e000341 10.1136/bmjoq-2018-000341 PMC6059260 30057962

[74] Palmer E, Richardson E, Newcombe H, Borg C-M. The F.R.I.D.A.Y.S. checklist—preparing our patients for a safe weekend. BMJ Qual Improv Rep 2015; 2:u660.w502 10.1136/bmjquality.u660.w502 PMC4663813 26734210

[75] Parwaiz H, Trew CA, Whitham R, Aliaga-Crespo B, Mitra A, Harding I. Improving the weekend spinal ward round at a major trauma centre. Br J Hosp Med (Lond) 2022;83:1–5 10.12968/hmed.2021.0209 35787168

[76] Pitcher M, Lin JT, Thompson G, Tayaran A, Chan S. Implementation and evaluation of a checklist to improve patient care on surgical ward rounds. ANZ J Surg 2016;86:356–360 25962703 10.1111/ans.13151

[77] Radhakrishnan NS, Lukose K, Cartwright R, Sleiman A, Matey N, Lim D et al Prospective application of the interdisciplinary bedside rounding checklist ‘TEMP’ is associated with reduced infections and length of hospital stay. BMJ Open Qual 2022;11:e002045 10.1136/bmjoq-2022-002045 PMC9723909 36588303

[78] Redley B, Campbell D, Stockman K, Barnes S. A mixed methods quality evaluation of structured interprofessional medical ward rounds. Internal Med J 2020;50:222–231 31069904 10.1111/imj.14330

[79] Rehder KJ, Uhl TL, Meliones JN, Turner DA, Smith PB, Mistry KP. Targeted interventions improve shared agreement of daily goals in the pediatric intensive care unit. Pediatr Crit Care Med 2012;13:6–10 21478796 10.1097/PCC.0b013e3182192a6c PMC3163112

[80] Sharma S, Peters MJ. ‘Safety by DEFAULT’: introduction and impact of a paediatric ward round checklist. Crit Care 2013;17:R232 24479381 10.1186/cc13055 PMC4028750

[81] Shirreff L, Husslein H, Lefebvre GG, Shore EM. Introduction of physician-nurse bedside rounding and ward task list to improve quality of care in gynaecology: prospective, single-blinded, Pre- and post-intervention study. J Obstet Gynaecol Can 2019;41:1108–1114 30686607 10.1016/j.jogc.2018.11.004

[82] Simon K, Sankara IR, Gioe C, Newcomb P. Including family members in rounds to improve communication in intensive care. J Nurs Care Qual 2021;36:25–31 32282508 10.1097/NCQ.0000000000000483

[83] Southwick F, Lewis M, Treloar D, Cherabuddi K, Radhakrishnan N, Leverence R et al Applying athletic principles to medical rounds to improve teaching and patient care. Acad Med 2014;89:1018–1023 24979169 10.1097/ACM.0000000000000278

[84] Spaner D, Caraiscos VB, Muystra C, Furman ML, Zaltz-Dubin J, Wharton M et al Use of standardized assessment tools to improve the effectiveness of palliative care rounds: a quality improvement initiative. J Palliat Care 2017;32:134–140 29096574 10.1177/0825859717740051

[85] Stroud MH, Moss MM, Gilliam CH, Honeycutt M, Frost M, Green JW. Introduction of a rounding sticker improves care and reduces infection rates in the pediatric intensive care unit (PICU). J Ark Med Soc 2012;109:114–117 23189772

[86] Sunkara P, Islam T, Bose A, Rosenthal GE, Chevli P, Jogu H et al Impact of structured interdisciplinary bedside rounding on patient outcomes at a large academic health centre. BMJ Qual Saf 2020;29:569–575 10.1136/bmjqs-2019-009936 PMC10189805 31810994

[87] Talia AJ, Drummond J, Muirhead C, Tran P. Using a structured checklist to improve the orthopedic ward round: a prospective cohort study. Orthopedics 2017;40:e663–e667 28504810 10.3928/01477447-20170509-01

[88] Thompson AG, Jacob K, Fulton J, McGavin CR. Do post-take ward round proformas improve communication and influence quality of patient care? Postgraduate Med J 2004;80:675–676 10.1136/pgmj.2003.016097 PMC1743139 15537856

[89] Torregrosa L, Ariza A, Villarreal L, Cabrera Vargas LF. Development and application of an inpatient traffic lights classification to improve the surgical ward round quality. Am J Surg 2022;223:1010–1012 34702490 10.1016/j.amjsurg.2021.10.012

[90] Trahan C, Hui AY, Binepal N. Standardization of rounds on a general paediatric ward: implementation of a checklist to improve efficiency, quality of rounds, and family satisfaction. Paediatr Child Health 2022;27:111–117 35599681 10.1093/pch/pxab080 PMC9113846

[91] Tranter-Entwistle I, Best K, Ianev R, Beresford T, McCombie A, Laws P. Introduction and validation of a surgical ward round checklist to improve surgical ward round performance in a tertiary vascular service. ANZ J Surg 2020;90:1358–1363 32356576 10.1111/ans.15899

[92] Urisman T, Garcia A, Harris HW. Impact of surgical intensive care unit interdisciplinary rounds on interprofessional collaboration and quality of care: mixed qualitative-quantitative study. Intensive Crit Care Nurs 2018;44:18–23 28865984 10.1016/j.iccn.2017.07.001

[93] Vukanic D, Kelly EG, Cleary SM. Does an orthopedic ward round pro forma improve inpatient documentation? J Patient Saf 2021;17:553–556 32168277 10.1097/PTS.0000000000000678

[94] Weiss CH, Moazed F, McEvoy CA, Singer BD, Szleifer I, Amaral LA et al Prompting physicians to address a daily checklist and process of care and clinical outcomes: a single-site study. Am J Respir Crit Care Med 2011;184:680–686 21616996 10.1164/rccm.201101-0037OC PMC3208596

[95] Wright DN . Does a post-take ward round proforma have a positive effect on completeness of documentation and efficiency of information management? Health Informatics J 2009;15:86–94 19474222 10.1177/1460458209102970

[96] Xu A, Chan LY, Abedin M, Sivapathasuntharam D. Use of a proforma to improve documentation of the post-take ward round and encourage initiation of the comprehensive geriatric assessment in the care of the older people’s service. Br J Hosp Med (Lond) 2021;82:1–6 10.12968/hmed.2020.0604 33512288

[97] Becker C, Gamp M, Schuetz P, Beck K, Vincent A, Hochstrasser S et al Effect of bedside compared with outside the room patient case presentation on patients’ knowledge about their medical care: a randomized, controlled, multicenter trial. Ann Intern Med 2021;174:1282–1292 34181449 10.7326/M21-0909

[98] Cavalcanti AB, Bozza FA, Machado FR, Salluh JIF, Campagnucci VP, Vendramim P et al Effect of a quality improvement intervention with daily round checklists, goal setting, and clinician prompting on mortality of critically ill patients: a randomized clinical trial. JAMA 2016;315:1480–1490 27115264 10.1001/jama.2016.3463

[99] Clarke-Pounder JP, Boss RD, Roter DL, Hutton N, Larson S, Donohue PK. Communication intervention in the neonatal intensive care unit: can it backfire? J Palliat Med 2015;18:157–161 24983892 10.1089/jpm.2014.0037

[100] Cox ED, Jacobsohn GC, Rajamanickam VP, Carayon P, Kelly MM, Wetterneck TB et al A family-centered rounds checklist, family engagement, and patient safety: a randomized trial. Pediatr 2017;139:e20161688 10.1542/peds.2016-1688 PMC5404725 28557720

[101] Donovan AK, Spagnoletti C, Rothenberger S, Corbelli J. The impact of residents sitting at the bedside on patient satisfaction during team rounds. Patient Educ Couns 2020;103:1252–1254 31866194 10.1016/j.pec.2019.12.013

[102] Ellison LM, Nguyen M, Fabrizio MD, Soh A, Permpongkosol S, Kavoussi LR. Postoperative robotic telerounding: a multicenter randomized assessment of patient outcomes and satisfaction. Arch Surg 2007;142:1177–1181 18086984 10.1001/archsurg.142.12.1177

[103] Finn KM, Metlay JP, Chang Y, Nagarur A, Yang S, Landrigan CP et al Effect of increased inpatient attending physician supervision on medical errors, patient safety, and resident education: a randomized clinical trial. JAMA Intern Med 2018;178:952–959 29868877 10.1001/jamainternmed.2018.1244 PMC6145715

[104] Jaberi AA, Zamani F, Nadimi AE, Bonabi TN. Effect of family presence during teaching rounds on patient’s anxiety and satisfaction in cardiac intensive care unit: a double-blind randomized controlled trial. J Educ Health Promot 2020;9:22 32154317 10.4103/jehp.jehp_417_19 PMC7034170

[105] Lienard A, Merckaert I, Libert Y, Bragard I, Delvaux N, Etienne A-M et al Transfer of communication skills to the workplace during clinical rounds: impact of a program for residents. PLoS One 2010;5:e12426 20865055 10.1371/journal.pone.0012426 PMC2928743

[106] Osborn R, Grossman M, Berkwitt A. The effect of sitting versus standing on family perceptions of family-centered rounds. Hosp Pediatr 2021;11:e313–e316 34607885 10.1542/hpeds.2021-005999

[107] Roberts RP, Blackwell SC, Brown KM, Pedroza C, Sibai BM, Tyson JE. Early compared with delayed physician rounds on patient satisfaction of postpartum women: a randomized controlled trial. Obstet Gynecol 2016;128:381–386 27400002 10.1097/AOG.0000000000001528

[108] Read J, Perry W, Rossaak JI. Ward round checklist improves patient perception of care. ANZ J Surg 2021;91:854–859 33459481 10.1111/ans.16543

[109] Eden E, Rothenberger SD, DeKosky A, Donovan AK. The safe discharge curriculum: how we standardized residents’ approach to hospital discharge. J Gen Intern Med 2018; 33(Suppl 1): 372

[110] McKenna HP, Ashton S, Keeney S. Barriers to evidence-based practice in primary care. J Adv Nurs 2004;45:178–189 14706003 10.1046/j.1365-2648.2003.02879.x

[111] Mohan DR, Kumar KS. A study on the satisfaction of patients with reference to hospital services. Int J Bus Econ Manag Res 2011;1:15–25

[112] Schoenfelder T, Klewer J, Kugler J. Determinants of patient satisfaction: a study among 39 hospitals in an in-patient setting in Germany. Int J Qual Health Care 2011;23:503–509 21715557 10.1093/intqhc/mzr038

[113] Manias E, Kusljic S, Wu A. Interventions to reduce medication errors in adult medical and surgical settings: a systematic review. Ther Adv Drug Saf 2020;11:2042098620968309 33240478 10.1177/2042098620968309 PMC7672746

[114] Donaldson MS, Corrigan JM, Kohn LT. *To Err is Human: Building a Safer Health System*. 2000. https://pubmed.ncbi.nlm.nih.gov/25077248/ (accessed 8 August 2024). 25077248

[115] National Institute for Health and Care Excellence . *Structured Ward Rounds, Emergency and Acute Medical Care in over 16s: Service Delivery and Organisation*. 2018. https://www.ncbi.nlm.nih.gov/books/NBK564911/ (accessed 27 July 2024). 33270407

[116] Stanhope N, Vincent C, Taylor-Adams SE, O'Connor AM, Beard RW. Applying human factors methods to clinical risk management in obstetrics. Br J Obstet 1997;104:1225–1232 10.1111/j.1471-0528.1997.tb10967.x 9386021

[117] Vincent C, Taylor-Adams S, Chapman EJ, Hewett D, Prior S, Strange P et al How to investigate and analyse clinical incidents: clinical risk unit and association of litigation and risk management protocol. BMJ 2000;320:777–781 10720366 10.1136/bmj.320.7237.777 PMC1117773

[118] Vaucher C, Bovet E, Bengough T, Pidoux V, Grossen M, Panese F et al Meeting physicians’ needs: a bottom-up approach for improving the implementation of medical knowledge into practice. Health Res Policy Syst 2016;14:49 27431911 10.1186/s12961-016-0120-5 PMC4949753

[119] Menon D, Stafinski T. Bridging the “know-do” gap in healthcare priority-setting: what role has academic research played? Healthc Manage Forum 2005;18:26–32 16509278 10.1016/S0840-4704(10)60066-X

