# Supplementary material for: Optimizing ward rounds: systematic review and meta-analysis of interventions to enhance patient safety
Source: Br J Surg. 2025 Apr 9;112(4):znaf041. doi: 10.1093/bjs/znaf041 (PMC11979594; doi:10.1093/bjs/znaf041)
Supplement: znaf041_Supplementary_Data [file znaf041_supplementary_data.docx]

**Title:** Optimising ward rounds: A Systematic Review and meta-analysis of interventions to enhance patient safety

**Authors:** Ellie C. Treloar BMedSci (Hons)^a^, Jesse D. Ey BHMS(Hons)^a^, Matheesha Herath MBBS^a^, Nicholas P.R. Edwardes MBBS ^a^, Suzanne Edwards BN^b^, Martin H. Bruening MS^a^, Guy J. Maddern PhD^a^

**Affiliations:**

^a^ Department of Surgery, The University of Adelaide, The Queen Elizabeth Hospital, 28 Woodville Road, Woodville, SA 5011, Australia.

^b^ School of Public Health, The University of Adelaide, Adelaide, SA, Australia

**Corresponding author.**

Professor Guy J. Maddern, Department of Surgery, The University of Adelaide, The Queen Elizabeth Hospital, 28 Woodville Road, Woodville, SA 5011, Australia.

**ORC ID:** 0000-0003-2064-181X

**Supplementary Materials - Index**

| **Supplementary Methods** |  |
| --- | --- |
| Appendix 1 - Search Terms | *Page 2* |
| Appendix 2 – Pre-formulated data extraction plan | *Page 3* |
| **Supplementary Results** |  |
| Appendix 3 – Included studies | *Page 4* |
| Appendix 4 – Excluded studies | *Page 4* |
| Appendix 5 – Forest plot (Checklist intervention and Hospital Length of Stay) | *Page 5* |
| Appendix 6 - Forest plot (Checklist intervention and ICU Mortality) | *Page 5* |
| Appendix 7 - Forest plot (Checklist intervention and inpatient mortality) | *Page 5* |
| Appendix 8 – Forest plots for meta-analyses (Significant documentation) | *Page 6* |
| Appendix 9 – Forest plots for meta-analyses (non-significant documentation) | *Page 6* |
| Appendix 10 – Checklist Intervention and Qualitative Outcomes | *Page 6* |
| Appendix 11 - Forest plot (Ward Round Structure Intervention and time spent rounding per patient) | *Page 7* |
| Appendix 12 - Forest plot (Ward Round Structure Intervention and time spent rounding in total) | *Page 7* |
| Appendix 13 - Forest plot (Ward Round Structure Intervention and 30-day readmission) | *Page 7* |
| Appendix 14 - Forest plot (Ward Round Structure Intervention and length of stay) | *Page 7* |
| Appendix 15 – Structure Intervention and Qualitative Outcomes | *Page 7* |
| Appendix 16 – ‘Other’ Intervention and Qualitative Outcomes | *Page 7* |
| **Supplementary Appendices** |  |
| Appendix 17 – Newcastle-Ottawa Scale for observational studies | *Page 8* |
| Appendix 18 – RoB2 for randomized control trials | *Page 8* |

**Supplementary Methods**

**Appendix 1 – Search Terms**

Database: Ovid MEDLINE(R) ALL <1946 to January 23, 2023>

Search Strategy:

--------------------------------------------------------------------------------

1 exp Education/ (890322)

2 exp Aviation/ (33594)

3 exp Computer Simulation/ (287057)

4 exp Teaching/ (92469)

5 Decision Support Systems, Clinical/ (9295)

6 Feedback/ (33276)

7 exp Documentation/ (960684)

8 (intervention* or tool* or checklist* or check-list* or template* or proforma* or ticklist* or tick-list* or instrument* or education* or coach* or train* or mentor* or program* or aid* or guide* or sterile cockpit or aviation or simulate* or model* or teach* or instruct* or structur* or (decision* adj3 support*) or feedback or feed back or document* or record*).ti,ab. (10831053)

9 or/1-8 (11842318)

10 Teaching Rounds/ (1438)

11 ((ward* or attending or grand or clinical or teaching or patient* or inpatient* or medical or physician* or doctor* or nurs* or team* or interdisciplinary or multi-d* or bed* or work or hybrid or daily) adj round*).ti,ab. (4759)

12 or/10-11 (5383)

13 outcome assessment, health care/ or patient outcome assessment/ or critical care outcomes/ or patient reported outcome measures/ or exp treatment outcome/ (1312139)

14 exp Medical Errors/ (121115)

15 Time Management/ (2987)

16 exp Communication/ (356871)

17 Morbidity/ (33707)

18 exp "Costs and Cost Analysis"/ (262232)

19 exp "Quality of Health Care"/ or Benchmarking/ or Health Equity/ or Program Evaluation/ (7998104)

20 "length of stay"/ or patient discharge/ or patient readmission/ (145700)

21 personal satisfaction/ or Job Satisfaction/ or Patient Satisfaction/ (137708)

22 Time-to-Treatment/ (9755)

23 exp Learning/ (430946)

24 formative feedback/ (1147)

25 safety/ or patient safety/ (66900)

26 efficiency/ (15325)

27 Program Evaluation/ or Work Performance/ (68204)

28 (outcome* or accura* or error* or interrupt* or time or communicat* or morbidit* or delay* or cost* or quality or (standard adj1 care) or discharge* or (adverse adj3 (event* or effect* or reaction*)) or readmi* or re-admi* or rehospital* or complication* or (length adj1 stay) or LOS or satisf* or learning* or safety or productiv* or efficienc* or effect* or performance or improv*).ti,ab. (15891178)

29 or/13-28 (19133643)

30 and/9,12,28 (2808)

**************************

Database: APA PsycInfo <1806 to January Week 4 2023>

Search Strategy:

--------------------------------------------------------------------------------

1 exp education/ (477612)

2 exp aviation/ (2783)

3 exp simulation/ (78093)

4 exp teaching/ (134882)

5 exp decision support systems/ (3654)

6 exp training/ (87695)

7 exp feedback/ (33414)

8 (intervention* or tool* or checklist* or check-list* or template* or proforma* or ticklist* or tick-list* or instrument* or education* or coach* or train* or mentor* or program* or aid* or guide* or sterile cockpit or aviation or simulate* or model* or teach* or instruct* or structur* or (decision* adj3 support*) or feedback or feed back or document* or record*).ti,ab. (5109518)

9 or/1-8 (5126291)

10 ((ward* or attending or grand or clinical or teaching or patient* or inpatient* or medical or physician* or doctor* or nurs* or team* or interdisciplinary or multi-d* or bed* or work or hybrid or daily) adj round*).ti,ab. (620)

11 treatment outcomes/ or patient reported outcome measures/ (39551)

12 errors/ (11150)

13 time management/ (1847)

14 exp communication/ (350295)

15 morbidity/ (6215)

16 exp "costs and cost analysis"/ (47958)

17 exp "quality of care"/ (14697)

18 exp program evaluation/ or employee productivity/ (25805)

19 hospital discharge/ (2900)

20 treatment duration/ (4537)

21 exp job satisfaction/ or client satisfaction/ (27703)

22 hospital discharge/ or psychiatric hospital readmission/ (3917)

23 exp learning/ (297073)

24 exp safety/ (34562)

25 (outcome* or accura* or error* or interrupt* or time or communicat* or morbidit* or delay* or cost* or quality or (standard adj1 care) or discharge* or (adverse adj3 (event* or effect* or reaction*)) or readmi* or re-admi* or rehospital* or complication* or (length adj1 stay) or LOS or satisf* or learning* or safety or productiv* or efficienc* or effect* or performance or improv*).ti,ab. (3157673)

26 or/11-25 (3338734)

27 and/9-10,26 (468)

***************************

Database: Embase <1974 to 2023 January 23>

Search Strategy:

--------------------------------------------------------------------------------

1 quality assessment tool/ (1454)

2 exp education/ (1623075)

3 aviation/ (8261)

4 simulation/ or exp computer simulation/ or exp predictive model/ (399174)

5 teaching/ (104568)

6 decision support system/ (26552)

7 training/ (104719)

8 exp feedback system/ (133230)

9 exp documentation/ (600496)

10 (intervention* or tool* or checklist* or check-list* or template* or proforma* or ticklist* or tick-list* or instrument* or education* or coach* or train* or mentor* or program* or aid* or guide* or sterile cockpit or aviation or simulate* or model* or teach* or instruct* or structur* or (decision* adj3 support*) or feedback or feed back or document* or record*).ti,ab. (13619897)

11 or/1-10 (14448258)

12 teaching round/ (939)

13 ((ward* or attending or grand or clinical or teaching or patient* or inpatient* or medical or physician* or doctor* or nurs* or team* or interdisciplinary or multi-d* or bed* or work or hybrid or daily) adj round*).ti,ab. (7609)

14 or/12-13 (8038)

15 treatment outcome/ or clinical outcome/ or critical care outcome/ or exp outcome assessment/ or patient-reported outcome/ (1918899)

16 diagnostic accuracy/ (292255)

17 exp medical error/ (169211)

18 time management/ (5720)

19 interpersonal communication/ (173460)

20 morbidity/ (399518)

21 exp "cost"/ or exp "health care cost"/ (389853)

22 health care quality/ or benchmarking/ or clinical effectiveness/ or health equity/ or nursing outcome/ or exp program evaluation/ or "quality of nursing care"/ (475475)

23 hospital discharge/ (169205)

24 exp adverse event/ (942712)

25 hospital readmission/ (92183)

26 exp complication/ (1466838)

27 "length of stay"/ (250721)

28 satisfaction/ or job satisfaction/ or patient satisfaction/ (267568)

29 time to treatment/ (24015)

30 treatment response time/ (3145)

31 learning/ (240755)

32 learning/ or collaborative learning/ or constructive feedback/ or experiential learning/ (246371)

33 safety/ or exp patient safety/ (412757)

34 productivity/ (48529)

35 program effectiveness/ (5152)

36 performance/ (35960)

37 (outcome* or accura* or error* or interrupt* or time or communicat* or morbidit* or delay* or cost* or quality or (standard adj1 care) or discharge* or (adverse adj3 (event* or effect* or reaction*)) or readmi* or re-admi* or rehospital* or complication* or (length adj1 stay) or LOS or satisf* or learning* or safety or productiv* or efficienc* or effect* or performance or improv*).ti,ab. (20157174)

38 or/15-37 (21637581)

39 and/11,14,38 (5653)

***************************

Database: Ovid Emcare <1995 to 2023 Week 03>

Search Strategy:

--------------------------------------------------------------------------------

1 quality assessment tool/ (322)

2 exp education/ (594430)

3 aviation/ (2079)

4 simulation/ or exp computer simulation/ or exp predictive model/ (77835)

5 teaching/ (49222)

6 decision support system/ (9304)

7 training/ (31086)

8 exp feedback system/ (33447)

9 exp documentation/ (140493)

10 (intervention* or tool* or checklist* or check-list* or template* or proforma* or ticklist* or tick-list* or instrument* or education* or coach* or train* or mentor* or program* or aid* or guide* or sterile cockpit or aviation or simulate* or model* or teach* or instruct* or structur* or (decision* adj3 support*) or feedback or feed back or document* or record*).ti,ab. (3367127)

11 or/1-10 (3578095)

12 teaching round/ (132)

13 ((ward* or attending or grand or clinical or teaching or patient* or inpatient* or medical or physician* or doctor* or nurs* or team* or interdisciplinary or multi-d* or bed* or work or hybrid or daily) adj round*).ti,ab. (2281)

14 or/12-13 (2334)

15 treatment outcome/ or clinical outcome/ or critical care outcome/ or exp outcome assessment/ or patient-reported outcome/ (371940)

16 diagnostic accuracy/ (70670)

17 exp medical error/ (38718)

18 time management/ (2561)

19 interpersonal communication/ (75422)

20 morbidity/ (86294)

21 exp "cost"/ or exp "health care cost"/ (103948)

22 health care quality/ or benchmarking/ or clinical effectiveness/ or health equity/ or nursing outcome/ or exp program evaluation/ or "quality of nursing care"/ (124097)

23 hospital discharge/ (38954)

24 exp adverse event/ (142654)

25 hospital readmission/ (14924)

26 exp complication/ (207644)

27 "length of stay"/ (45851)

28 satisfaction/ or job satisfaction/ or patient satisfaction/ (98240)

29 time to treatment/ (2898)

30 treatment response time/ (191)

31 learning/ (93818)

32 learning/ or collaborative learning/ or constructive feedback/ or experiential learning/ (96351)

33 safety/ or exp patient safety/ (120016)

34 productivity/ (14168)

35 program effectiveness/ (1522)

36 performance/ (5877)

37 (outcome* or accura* or error* or interrupt* or time or communicat* or morbidit* or delay* or cost* or quality or (standard adj1 care) or discharge* or (adverse adj3 (event* or effect* or reaction*)) or readmi* or re-admi* or rehospital* or complication* or (length adj1 stay) or LOS or satisf* or learning* or safety or productiv* or efficienc* or effect* or performance or improv*).ti,ab. (4474757)

38 or/15-37 (4770453)

39 and/11,14,38 (1475)

***************************

**Appendix 2 – Data Extraction Plan**

| **Covidence ID** | **Study ID** | **Title** | **Reviewer Name** | **Title** | **Author and year published** | **Journal** | **Country** | **Study Design** | **Specialty** |
| --- | --- | --- | --- | --- | --- | --- | --- | --- | --- |
|  |  |  |  |  |  |  |  |  |  |
| **Aim** | **Start and End Date; Duration** | **Setting** | **Study funding sources** | **Possible conflicts of interest for authors** | **Population description** | **Inclusion Criteria** | **Exclusion Criteria** | **Method of recruitment of participants** | **Total Number of Participants recruited** |
|  |  |  |  |  |  |  |  |  |  |
| **Participants excluded (with reasons)** | **Total number of participants included in analysis** | **Population demographics** | **Description of intervention (s)** | **How was the intervention designed** | **Design of intervention** | **How was it implemented** | **Method of recruitment** | **Description of intervention (s)** | **How was the intervention designed** |
|  |  |  |  |  |  |  |  |  |  |
| **Design of intervention** | **How was it implemented** | **Method of recruitment** | **Description of intervention (s)** | **How was the intervention designed** | **Design of intervention** | **How was it implemented** | **Method of recruitment** | **Effects measured** | **Measuring points of outcomes** |
|  |  |  |  |  |  |  |  |  |  |
| **Intended outcomes to be measured in the study** | **Intended statistical Analysis to be used.** | **Tools to assess effects** | **Number of rounds / number of patients** | **Population 1** | **Population 2** | **Population 3** | **Outcome category 1** | **Results** | **Outcome Category 2** |
|  |  |  |  |  |  |  |  |  |  |
| **Outcome Category 3** | **Results** | **Outcome Category 4** | **Results** | **Outcome Category 5** | **Results** | **Outcome Category 6** | **Results** | **Were all intended outcomes reported?** | **Were additional outcomes reported?** |
|  |  |  |  |  |  |  |  |  |  |
| **Strengths** | **Weaknesses** | **Lay person summary of the study** | **Any additional comments** |  |  |  |  |  |  |

**Supplementary Results**

**Appendix 3 – Summary of included studies**

| **Study ID** | **Specialty/Specialties** | **Intervention** | **Sample Size/ Demographics** |
| --- | --- | --- | --- |
| Abraham 2019 | Family Medicine | Checklist  (Paper based) | 169 |
| AcalJimenez 2018 | Pediatrics | Structure  (Establishing Explicit Roles) | 213 |
| Alazzawi 2016 | Trauma and Orthopedics | Checklist  (Paper based proforma) | 60 |
| Al-Mahrouqi 2013 | General Surgery | Checklist  (Paper based proforma) | 211 |
| Armstrong 2022 | Specialist Stroke Unit | Checklist  (Paper based proforma) | 206 |
| Banfield 2015 | Acute surgical unit (ASU) | Checklist  (Paper based) | 146 |
| Becker 2021 | General Medicine | Structure  (Bedside or Outside) | 1092 |
| Blucher 2014 | Acute Surgical Care | Checklist  (Paper based) | 100 |
| Boland 2015 | Medical Assessment Unit | Checklist  (Paper based proforma) | 106 |
| Brown 2021 | Respiratory Medicine | Checklist  (Paper based proforma) | 27 |
| Brown 2019 | Trauma and orthopaedic surgery | Structure  (Communication survey) | 281 |
| Byrd 2018 | Pediatrics | Structure  + Education  (Mobile Devices) | 76 |
| Cao 2016 | Medical Intensive Care Unit | Structure  (Information to discuss, roles allocated) | 665 |
| Cavalcanti 2016 | Intensive Care Unit | Checklist (Paper based) | 13638 |
| Chow 2019 | Emergency Medicine | Structure  (Specific format, involve certain team members, occurs bedside) | 672 |
| Christensen 2017 | Obstetrics | Structure  (Specific questions and order to follow) | 150 |
| Christianson 2022 | Pediatrics | Structure  (Combined, multiple factors eg. rationale sharing, data feedback, standardisation, checklists) | 700 |
| Cifra 2019 | Pediatrics | Checklist (Paper based) | 444 |
| Clarke-Pounder 2015 | Pediatrics | Structure  (Decision Making Tool) | 55 |
| Clark 2019 | Pediatrics | Checklist (Paper based) | 2707 |
| Conroy 2015 | ICU | Checklist (Electronic) | 293 |
| Cox 2017 | Paediatrics | Checklist (Paper based) | 298 |
| Crowson 2016 | Otolaryngology | Structure  (Tablet use) | N/A |
| DeBie 2021 | ICU | Checklist (Electronic) | 401 |
| Dhillon 2011 | Surgery (incl. general surgery, vascular surgery, plastic surgery and neurosurgery). | Checklist (Paper based) | 198 |
| Dodek 2003 | ICU | Structure  (Worksheet to guide responsibilities) | 345 |
| Dolan 2016 | General Surgery | Checklist  (Paper based proforma) | 97 |
| Donovan 2020 | Internal Medicine | Structure  (Sitting vs Standing) | 347 |
| Duxbury 2013 | Trauma/ orthopaedic surgery | Checklist (Proforma; Paper and electronic) | 100 |
| Eden 2022 | Academic medical centre | Education + Checklist (Paper based) | 160 |
| Efune 2018 | Paediatrics | Checklist (Paper based) | 582 |
| Ellison 2007 | Urology | Structure  (Telerounding Robot) | 270 |
| Escamilla-Ocanas 2022 | Neurocritical Care Unit (NCCU) | Checklist (Electronic) | 1062 |
| Feinman 2022 | General Surgery | Structure  (Lean rounds) | 22 |
| Finn 2018 | General Medicine | Structure  (Increased Supervision) | 1303 |
| Fleischmann 2015 | Neurology | Structure  (Tablet Computers) | 164 |
| Galloway 2022 | Acute Medicine | Checklist (Paper based) | 325 |
| Gilliland 2018 | Urology | Checklist (Paper based) | 45 |
| Glick 2022 | Pediatrics | Checklist (standardised template) + teaching | 2447 |
| Hale 2015 | Gastroenterology | Checklist (Paper based) | N/A |
| Harman 2019 | Paediatrics, Internal Medicine | Education | 527 |
| Jaberi 2020 | Cardiac Intensive Care Unit | Structure  (Encouraged family presence) | 60 |
| Johnston 2022 | Surgery (included colorectal, orthopaedics, vascular, urology, and upper GI teams) | Checklist (Paper based) | 189 |
| Justice 2016 | Cardiac ICU | Structure  (Writing down goals + reading back) | N/A |
| Kamara 2006 | Medical assessment unit | Checklist (Paper based) | 226 |
| Kashyap 2021 | Paediatric Intensive Care | Checklist (Paper based and electronic) | 735 |
| Keller 2018 | General Medicine | Checklist (Electronic) | 62 |
| Khan 2018 | Pediatrics | Structure | 3106 |
| Koumoullis 2020 | Plastic Surgery | Checklist (Paper based proforma) | 145 |
| Krishnamohan 2019 | Surgical (including urology and vascular surgery) | Checklist (Paper based) | 133 |
| Lepee 2012 | Pediatrics | Checklist (Paper based) | 227 |
| Licata 2011 | Pediatrics | Structure  (PICU rounding model) | N/A |
| Lienard 2010 | Oncology, Gynaecology, Surgery, Gastroenterology | Education | 88 |
| Ludley 2023 | Stroke | Checklist (Paper based) | 142 |
| Murphy 2015 | General Medicine | Checklist (Paper based) | 200 |
| Nassikas 2020 | ICU | Checklist | N/A |
| Newnham 2015 | Pediatrics | Checklist (Paper based) | 200 |
| Ng 2018 | General Surgery | Checklist (Paper based) | 256 |
| Osborn 2021 | Pediatrics | Structure  (Sitting vs standing) | 103 |
| Palmer 2014 | Geriatrics | Checklist (Paper based) | 50 |
| Parwaiz 2022 | Trauma/spinal | Checklist (Paper based) | 219 |
| Pitcher 2016 | General Surgery | Checklist (Paper based) | 314 |
| Radhakrishanan 2022 | Not stated | Checklist (Paper based) | N/A |
| Read 2021 | Surgery | Checklist (Electronic) | 155 |
| Redley 2019 | General Medicine | Structure | 317 |
| Rehder 2012 | Pediatrics | Checklist (Paper based) + Structure | 736 |
| Roberts 2016 | Obstetrics and gynaecology | Structure  (Early vs late rounding) | 152 |
| Sharma 2013 | Pediatrics | Checklist (Paper based) | 103 |
| Shirreff 2019 | Obstetrics and Gynaecology | Structure | 223 |
| Simon 2021 | Neurosurgical ICU | Structure | 123 |
| Southwick 2014 | Internal Medicine | Other  (Athletic principles) | 780 |
| Spaner 2017 | Palliative Care Unit (PCU) | Education | 21 |
| Stroud 2012 | Paediatric intensive care | Checklist (Paper based) | 2930 |
| Sunkara 2020 | Hospital Medicine Unit | Structure | 2221 |
| Talia 2017 | Orthopaedics | Checklist (Paper based) | 200 |
| Thompson 2004 | Not stated | Checklist (Paper based proforma) | 190 |
| Torregrosa 2022 | General surgery | Other (Traffic Light System) | N/A |
| Trahan 2022 | Pediatrics | Checklist (Paper based) | 59 |
| Tranter-Entwistle 2020 | Vascular Surgery | Checklist (Paper based) | 233 |
| Urisman 2018 | General Surgery | Structure | 169 |
| Vukanic 2021 | Orthopaedics | Checklist (Paper based proforma) | 60 |
| Weiss 2011 | Intensive care | Checklist (Paper based) | 265 |
| Wright 2009 | Not stated | Checklist (Paper based) | 170 |
| Xu 2021 | Acute medicine (Geriatric section) | Checklist (Electronic) | 124 |

**Appendix 4 – Excluded studies and reason for exclusion**

| **Author** | **Journal** | **Title** | **Reason** |
| --- | --- | --- | --- |
| Abbass 2016 | Pediatric Critical Care Medicine | Impact of introduction of rounding checklist in pediatric intensive care unit of a developing country: A quality improvement project | Abstract / E - Poster |
| Adams 2016 | Palliative Medicine | Resuscitation decisions; a sticky situation | Abstract / E - Poster |
| Adamson 2010 | Critical Care | Improving team work during bedside rounds: Using daily goals and best practices | Abstract / E - Poster |
| Alamri 2016 | ANZ journal of surgery | Surgical ward round checklist: does it improve medical documentation? A clinical review of Christchurch general surgical notes | No intervention / baseline / comparator |
| Algaze 2016 | Pediatrics | Use of a Checklist and Clinical Decision Support Tool Reduces Laboratory Use and Improves Cost | Wrong outcomes |
| Allenbaugh 2017 | Journal of General Internal Medicine | A communication intervention aimed at medicine doctors and nurses improves patient satisfaction scores | Abstract / E - Poster |
| Alsos 2011 | Methods of information in medicine | Doctors' concerns of PDAs in the ward round situation. Lessons from a formative simulation study | Abstract / E-Poster |
| Amin 2012 | Journal of the Royal Society of Medicine | Why patients need leaders: introducing a ward safety checklist | Wrong Study Design: No intervention, baseline or comparator |
| Archibald 2022 | Journal of General Internal Medicine | NET ROUNDING: A NOVEL APPROACH TO EFFICIENT AND EFFECTIVE ROUNDS FOR THE MODERN CLINICAL LEARNING ENVIRONMENT | Wrong outcomes |
| Arguello 2014 | Critical Care Medicine | Evaluation of rounding structure in a medical ICU before and after electronic rounding tool implementation | Abstract / E-Poster |
| Armstrong 2021 | British Journal of Surgery | A proforma to improve documentation of surgical ward rounds | Abstract / E-Poster |
| Arora 2021 | International Journal of Stroke | A post take ward round check-list for venous thromboembolism prevention in stroke patients: A quality improvement project | Abstract / E-Poster |
| Asmat 2018 | International Journal of Surgery | Improving the quality of urology ward round documentation | Abstract / E-Poster |
| Austin 2020 | Journal of the American Association of Nurse Practitioners | Evaluation of a nurse practitioner-led project to improve communication and collaboration in the acute care setting | Wrong outcomes |
| Aydin 2014 | Minerva chirurgica | Comparison between electronic method and conventional method recording and follow-up of general surgery ward-round notes taken | Wrong outcomes |
| Azorin-Samper 2020 | Ciclo de mejora del proceso asistencial en una sala de hospitalizacion de cirugia general | A quality improvement cycle of the care process in a general surgery hospitalization ward | Wrong outcomes |
| Barker 2015 | Journal of General Internal Medicine | Bring it to the bedside: Tablet computers increase team patient interactions | Abstract / E-Poster |
| Barton 2012 | Journal of pediatric nursing | New knowledge, innovations, and improvement in a Magnet R Children's Hospital Cardiac Center | Wrong population |
| Baryeh 2018 | British journal of hospital medicine | Does a daily consultant ward round affect the outcomes of orthopaedic patients? | Abstract / E-Poster |
| Basic 2018 | Clinical interventions in aging | Structured interdisciplinary bedside rounds, in-hospital deaths, and new nursing home placements among older inpatients | Wrong intervention |
| Basic 2021 | Journal of the American Geriatrics Society | Twice-Weekly Structured Interdisciplinary Bedside Rounds and Falls among Older Adult Inpatients | Wrong intervention |
| Basnet 2013 | Critical Care Medicine | Effect of protocol mandating discussion about ICU status change in a pediatric ICU | Abstract / E-Poster |
| Beatriz 2010 | Academic pediatrics | Applying lean initiatives to inpatient rounds to improve discharge delays | Abstract / E-Poster |
| Berry 2019 | British Journal of Surgery | Transforming surgical ward rounds with proforma-based standardised entries | Abstract / E - Poster |
| Blankenburg 2016 | Journal of General Internal Medicine | Got SDM?: A multimodal intervention to improve shared decision-making during inpatient rounds on medicine and pediatric services | Abstract / E - Poster |
| Blankenburg 2016 | Academic pediatric | Shared decision making: A multi-site educational bundle improves patient engagement and communication during inpatient rounds | Abstract / E-Poster |
| Bohrn 2014 | Academic Emergency Medicine | Two for one: Residency leadership team rounding to assess/improve the patient experience and gain emergency medicine resident patient feedback | Abstract / E-Poster |
| Boissaud-Cooke 2018 | British journal of neurosurgery | Development of a ward round checklist to improve patient safety and flow on the neurosurgical ward | Abstract / E-Poster |
| Borich 2015 | Biology of Blood and Marrow Transplantation | Rounding with purpose: Improving communication, safety and collaboration with nurse driven, multidisciplinary family rounds | Abstract / E-Poster |
| Boushehri 2013 | Medical education | Effects of morning report case presentation on length of stay and hospitalisation costs | Wrong setting |
| Boydston 2018 | JBI Database of Systematic Reviews and Implementation Reports | Use of a standardized care communication checklist during multidisciplinary rounds in pediatric cardiac intensive care: a best practice implementation project | Wrong population |
| Brewster 2017 | International Journal of Surgery | Medipack: Refining the plastic surgery ward round | Abstract / E-Poster |
| Brodie 2014 | Intensive Care Medicine | Use of a seven question checklist to improve ICU ward round reliability and patient safety | Abstract / E - Poster |
| Brooks 2022 | Intensive Care Medicine Experimental | Documentation of Microbiology Ward Round Pre and Post CareFlow Implementation on the ICU at Buckinghamshire Healthcare Trust | Abstract / E - Poster |
| Brown 2020 | The Journal of nursing administration | Standardizing Multidisciplinary Rounds: Creation of an Efficient and Effective Process to Care for the Critically Ill | Wrong population: nurses only |
| Brown 2021 | BJS open | Quality improvement project: Improving documentation and junior doctor confidence on covid-19 ward rounds using a ward round pro forma | Abstract / E - Poster |
| Bullock 2019 | International Journal of Clinical Pharmacy | The impact of a pharmacist on post-take ward round prescribing and medication appropriateness | Wrong intervention |
| Butler 2018 | American Journal of Respiratory and Critical Care | Device utilization before and after implementation of a checklist on multi-disciplinary rounds in a community hospital | Abstract / E-Poster |
| Caprirolo 2013 | Critical Care Medicine | Checklists improve compliance and quality health care delivery in the pediatric ICU: A pilot study | Abstract / E-Poster |
| Carlos 2015 | Annals of the American Thoracic Society | Intensive care unit rounding checklist implementation. Effect of accountability measures on physician compliance | Wrong outcomes |
| Carroll 2008 | Qualitative health research | Reshaping ICU ward round practices using video-reflexive ethnography | Wrong outcomes |
| Chan 2022 | BMC medical education | NET Rounding: a novel approach to efficient and effective rounds for the modern clinical learning environment | Wrong outcomes |
| Chapman 2021 | Intensive & critical care nursing | Benefits of collaborative patient care rounds in the intensive care unit | Wrong intervention |
| Chowdhury 2018 | American Journal of Respiratory and Critical Care | Structured interprofessional bedside rounds increase engagement in a medical intensive care unit | Abstract / E-Poster |
| Clay-Williams 2018 | Journal of hospital medicine | Improving Teamwork and Patient Outcomes with Daily Structured Interdisciplinary Bedside Rounds: A Multimethod Evaluation | Wrong intervention |
| Conroy-Smith 2011 | The clinical teacher | Learning safe prescribing during post-take ward rounds | No intervention / baseline / comparator |
| Cornell 2014 | The Journal of nursing administration | Improving situation awareness and patient outcomes through interdisciplinary rounding and structured communication | Wrong setting |
| Counihan 2016 | American journal of medical quality : the official journal of the American College of Medical Quality | Surgical Multidisciplinary Rounds: An Effective Tool for Comprehensive Surgical Quality Improvement | Wrong intervention |
| Coyle 2022 | British Journal of Surgery | The role of proformas in improving the documentation of surgical ward rounds | Abstract / E-Poster |
| Cummings 2013 | Critical Care Medicine | Reduce pediatric intensive care unit (PICU) rounds inefficiency through a preround huddle | Abstract / E-Poster |
| Curley 1998 | Medical care | A firm trial of interdisciplinary rounds on the inpatient medical wards: an intervention designed using continuous quality improvement | Wrong population |
| Curtis 2019 | British Journal of Surgery | Improving Surgical Ward Round documentation: Looped Audit | Abstract / E - Poster |
| Curtis 2013 | Journal of Psychiatric Intensive Care | Creating a high quality consultant led psychiatric intensive care unit multidisciplinary team ward round | No intervention / comparator / baseline |
| Davies 2017 | American Journal of Respiratory and Critical Care Medicine | Outcomes of the implementation of a daily ICU checklist during rounds in a resource poor ICU setting | Abstract / E - Poster |
| Delicata 2015 | International Journal of Stroke | Introduction of ward round checklist in acute stroke wards in royal Hallamshire Hospital, Sheffield | Abstract / E - Poster |
| Dewson 2020 | Postgraduate medical journal | Surgical ward round proforma can improve documentation and efficiency of ward rounds | Letter |
| Dhariwal 2021 | Gynaecology | Scrutinising the scribble; a quality improvement project of gynaecology inpatient documentation | Abstract / E-Poster |
| Diviatia 2013 | Journal of General Internal Medicine | Leaning towards patient-centered teaching rounds | Abstract / E - Poster |
| Duan 2014 | American Journal of Respiratory and Critical Care Medicine | Use of a daily goals checklist for morning intensive care unit rounds: A mixed-methods study | Abstract / E - Poster |
| Dunn 2017 | Journal of hospital medicine | The Impact of Bedside Interdisciplinary Rounds on Length of Stay and Complications | Wrong setting |
| Eissa 2022 | Archives of Disease in Childhood | Ward Round Sheet | Abstract / E-Poster |
| Eulmesekian 2017 | Pediatricos | Implementation of a checklist to increase adherence to evidence-based practices in a single pediatric intensive care unit | Wrong Study design |
| Evans 2021 | British Journal of Surgery | Using urology specific ward round sheets to improve clinical documentation | Abstract / E-Poster |
| Fernandes 2017 | BMJ quality improvement reports | Electronic Printed Ward Round Proformas: Freeing Up Doctors' Time | Wrong Study design |
| Ford 2017 | The clinical teacher | Simulated ward round: reducing costs, not outcomes | Compares simulation |
| Ganesan 2011 | Pediatric Critical Care Medicine | Development of structured rounding checklist in the pediatric intensive care unit | Abstract / E-Poster |
| Ganesan 2011 | Critical Care Medicine | Structured rounding checklist improves quality of care in the PICU | Abstract / E-Poster |
| Ganesan 2017 | Pediatric quality & safety | WE CARE 4 KIDS: Use of a Rounding Tool in the Pediatric Intensive Care Unit | Wrong population |
| Garr 2021 | Archives of Disease in Childhood | Implementation of a neonatal daily review sheet on a post-natal ward | Abstract / E - Poster |
| Gausvik 2017 | Journal of the American Geriatrics Society | Significant reduction in length of stay and readmission rate over 3 years on an ace unit optimized with daily structured interdisciplinary bedside rounds | Abstract / E-Poster |
| Gausvik 2015 | Journal of multidisciplinary healthcare | Structured nursing communication on interdisciplinary acute care teams improves perceptions of safety, efficiency, understanding of care plan and teamwork as well as job satisfaction | Wrong outcomes |
| Geva 2021 | Pediatric critical care medicine : a journal of the Society of Critical Care Medicine and the World Federation of Pediatric Intensive and Critical Care Societies | eSIMPLER: A Dynamic, Electronic Health Record-Integrated Checklist for Clinical Decision Support During PICU Daily Rounds | Wrong comparator |
| Giannotti 2022 | Journal of the American Medical Directors Association | Effect of Geriatric Comanagement in Older Patients Undergoing Surgery for Gastrointestinal Cancer: A Retrospective, Before-and-After Study | Wrong intervention: introduction of co-management with geriatric team |
| Gidwani 2021 | British Journal of Surgery | Improving documentation during a general surgical ward round | Abstract / E - Poster |
| Gonzalo 2010 | Journal of General Internal Medicine | The return of bedside rounds: an educational intervention | Wrong outcomes |
| Goolsarran 2020 | Journal of General Internal Medicine | Outcomes of a Resident-Led Early Hospital Discharge Intervention | Wrong intervention |
| Graham-Glover 2017 | Open Forum Infectious Diseases | The intern intervention: The effect of daily rounding on reducing indwelling urinary catheter device utilization | Abstract / E - Poster |
| Gray 2020 | The clinical teacher | Structuring ward rounds to enhance education | No intervention / baseline / comparator |
| Green 2014 | BMJ quality improvement reports | Ward round documentation in a major trauma centre: can we improve patient safety? | Wrong outcomes |
| Grey 2015 | BJU international | 'Registrar of the Week' and a bespoke ward round checklist optimise the quality of urology in-patient care | Abstract / E - Poster |
| Groome 2017 | Critical Care | The electronic documentation of FAASTHUG on the intensive care unit ward round | Abstract / E - Poster |
| Groome 2016 | Journal of the Intensive Care Society | Documentation of FASTHUG-improving the standard of care | Abstract / E-Poster |
| Grunewald 2020 | Patient Education and Counseling | Improving physicians' surgical ward round competence through simulation-based training | Compares simulation |
| Gupta 2016 | Journal of General Internal Medicine | Pilot study to examine the effect of a simple intervention during rounds on lab-ordering practices among inpatient house-staff teams-the 'labs tomorrow' study | Abstract / E - Poster |
| Hache-Marliere 2017 | American Journal of Respiratory and Critical Care | Certain rounding model with software prompting improves patient rounds and process of care in ICU | Abstract / E-Poster |
| Hacon 2010 | Intensive Care Medicine | Daily goals augment effective communication in the multidisciplinary ICU team | Abstract / E-Poster |
| Haeffs 2012 | Critical Care Medicine | Impact of a daily rounding checklist on device utilization in the pediatric intensive care unit (PICU) | Abstract / E-Poster |
| Harbham 2021 | Intensive Care Medicine Experimental | Improving the quality of the Intensive Care Unit (ICU) microbiology ward round documentation through the introduction of a microbiology ward round proforma | Abstract / E - Poster |
| He 2015 | Critical Care Medicine | Teamstepps rounding improvement project: An integrated rounding style | Abstract / E-Poster |
| He 2016 | Surgical infection | Benefit of TeamSTEPPS Rounding Improvement Project on Infection-Related Monitoring | Wrong intervention |
| Hemadasa 2020 | British Journal of Surgery | Surgical ward round toolkit | Abstract / E-Poster |
| Henkin 2016 | Journal of multidisciplinary healthcare | Improving nurse-physician teamwork through interprofessional bedside rounding | Wrong outcomes |
| Henning 2017 | Journal of the American Geriatrics Society | Structured interdisciplinary bedside rounds (SIBR) for skilled nursing patients reduce hospital readmissions and length of stay | Abstract / E - Poster |
| Herring 2011 | Clinical Governance | Implementation of a considerative checklist to improve productivity and team working on medical ward rounds | Wrong study design |
| Herring 2011 | Clinical medicine | Quality and safety at the point of care: how long should a ward round take? | No intervention / comparator / baseline |
| Hockenberry 2022 | Journal of Burn Care and Research | A Multi-pronged Approach to Improving Communication, Collaboration and Outcomes in the Burn ICU | Abstract / E - Poster |
| Horton 2015 | BMJ quality improvement reports | Improving documentation of physical health investigations in an adolescent mental health inpatient unit | No intervention |
| Hou 2019 | Critical Care | Effect of checklist for early recognition and treatment of acute illness for morning rounds on the prognosis of critically ill patients | Abstract / E - Poster |
| Hou 2019 | Zhonghua nei ke za zhi | [The application of checklist in ward rounds on the prognosis of critically ill patients] | Wrong outcomes |
| Hough 2009 | Critical Care Medicine | Effect of implementing bedside goal sheets on time to complete tasks in a pediatric intensive care unit | Abstract / E – Poster |
| Hussain 2011 | Colorectal Disease | The weekend handover stamp; improving patient safety and quality of care | Abstract / E - Poster |
| Huynh 2017 | Australian health review : a publication of the Australian Hospital Association | Structured interdisciplinary bedside rounds do not reduce length of hospital stay and 28-day re-admission rate among older people hospitalised with acute illness: an Australian study | Wrong intervention |
| Johns 2021 | British Journal of Surgery | The introduction of standardised ward round notes in the Acute Surgical Unit | Abstract / E - Poster |
| Johnston 2018 | Annals of surgery | Improving the Quality of Ward-based Surgical Care With a Human Factors Intervention Bundle | Wrong setting: Ward setting but not rounds |
| Jones 2019 | Age and Ageing | A junior doctor intervention to improve patient experience on geriatric wards | Abstract / E - Poster |
| Kajouj 2020 | British Journal of Surgery | Implementation of structured ward round documentation to enhance patients' care | Abstract / E - Poster |
| Kapoor 2010 | Critical care explorations | Impact of Geographical Cohorting in the ICU: An Academic Tertiary Care Center Experience | Wrong intervention |
| Kane 2018 | Journal of hospital medicine | Lean-Based Redesign of Multidisciplinary Rounds on General Medicine Service | Wrong intervention |
| Kapp 2014 | American Journal of Respiratory and Critical Care Medicine | A modified checklist to improve standard care orders in the medical intensive care unit | Abstract / E - Poster |
| Kapriniotis 2022 | British Journal of Surgery | 'smart phrase' ward rounds: Do they improve documentation and efficiency? | Abstract / E - Poster |
| Kawai 2018 | Critical Care Medicine | PICU liberation collaborative: Deescalation of critical care through daily rounding checklist | Abstract / E - Poster |
| Kawai 2018 | Critical Care Medicine | PICU liberation collaborative: Bundle to eliminate delirium improves ICU culture and outcomes | Abstract / E - Poster |
| Khalaf 2022 | Interactive journal of medical research | Education During Ward Rounds: Systematic Review | Review |
| Khaleeq 2022 | British Journal of Surgery | Making an Effective Ward Round Model in the Department of Trauma and Orthopaedic Surgery for University Hospitals of Birmingham Trust | Abstract / E - Poster |
| Kilsby 2017 | Age and Ageing | Improving the recording of comprehensive geriatric assessment and safety factors by use of a checklist | Abstract / E - Poster |
| Kocolas 2018 | Pediatrics | Finding the middle path: Balancing collaborative patient care with high-yield medical education | Abstract / E - Poster |
| Kotecha 2015 | American Journal of Respiratory and Critical Care Medicine | Reducing unnecessary lab tests in the MICU by incorporating a guideline in daily ICU team rounds | Abstract / E - Poster |
| Krauyter 2014 | Medical Teacher | Improving ward round skills | Wrong outcomes |
| Krishnan 2014 | BMJ Publishing Group | Transforming ward rounds | Abstract / E - Poster |
| LaRosa 2019 | American journal of perinatology | The Effect of Wearing White Coats on Patients' Appreciation of Physician Communication during Postpartum Rounds: A Randomized Controlled Trial | Abstract / E - Poster |
| Lattey 2017 | European Journal of Heart Failure | An audit exploring the effectiveness in medical data recording on implementation of an inpatient heart failure proforma | Abstract / E - Poster |
| Lee 2016 | International Journal of Surgery | Safe surgical ward rounds, a completed quality improvement cycle | Abstract / E - Poster |
| Lenathen 2018 | Pediatric Critical Care Medicine | The importance of being valued: Introduction of a novel ward round checklist in a paediatric intensive care unit | Abstract / E - Poster |
| Leung 2017 | Canadian Journal of Cardiology | Development and implementation of a bedside rounds checklist in a cardiac ICU (CICU) | Abstract / E - Poster |
| Liao 2021 | British Journal of Surgery | A closed loop audit looking at the use of a checklist in surgical ward rounds to improve documentation. Can we improve patient safety? | Abstract / E - Poster |
| Lindemuth 2018 | Critical Care Medicine | Define, detect, and protect: Reducing distractions in the trauma intensive care unit | Abstract / E - Poster |
| Lopez 2019 | Pediatric quality & safety | Impacting Satisfaction, Learning, and Efficiency Through Structured Interdisciplinary Rounding in a Pediatric Intensive Care Unit: A Quality Improvement Project | Wrong intervention |
| Lyckhage 2014 | Cerebrovascular Diseases | The involving interdisciplinary ward round; impact on patient experience in stroke rehabilitation | Abstract / E - Poster |
| Macklin 2011 | QJM | Does the use of a pro forma improve documentation of the post-take ward round? | Abstract / E - Poster |
| Maran 2022 | Revista gaucha de enfermagem | Multiprofessional round with checklist: association with the improvement in patient safety in intensive care | Wrong outcomes |
| Maran 2022 | Revista brasileira de enfermagem | Effects of multidisciplinary rounds and checklist in an Intensive Care Unit: a mixed methods study | Wrong outcomes |
| MarcOverhage 1996 | Archives of Internal Medicine | Computer reminders to implement preventive care guidelines for hospitalized patients | Wrong intervention |
| Marco 2016 | Canadian Journal of Cardiology | Collaboratively improving cardiology in-patient flow | Abstract / E - Poster |
| Mastalerz 2018 | Journal of General Internal Medicine | Teaching communication on inter professional bedside rounds: Improving resident performance on accountable care units | Abstract / E - Poster |
| Mastalerz 2018 | Journal of hospital medicine | Using an intensive feedback curriculum to impact medical resident interprofessional teamwork behaviors and attitudes | Abstract / E - Poster |
| Mastalerz 2020 | Journal of General Internal Medicine | Engaging stakeholders to improve healthcare team communication | Abstract / E - Poster |
| McClintock-Tiongco 2015 | European Geriatric Medicine | The use of a proforma to improve the quality of ward round documentation in care of the elderly medical wards | Abstract / E - Poster |
| McFarlane 2022 | European Geriatric Medicine | Use of a documentation tool to support geriatric inpatient IV access care | Abstract / E - Poster |
| McKelvie 2016 | International journal for quality in health care : journal of the International Society for Quality in Health Care | A PICU patient safety checklist: rate of utilization and impact on patient care | No intervention / comparator / baseline |
| McLoughlin 2021 | British Journal of Surgery | Improving the urology post take ward round with best clinical practice and a governance checklist. Quality improvement project within belfast health and social care trust 2020 | Abstract / E - Poster |
| Michel 2016 | Colorectal Disease | Perioperative patient outcomes following emergency colorectal resections after introduction of twice-daily acute consultant ward rounds | Abstract / E - Poster |
| Miller 2018 | Critical Care Medicine | Implementation of a daily goals tool improves team discussion of quality and safety practices | Abstract / E - Poster |
| Mohan 2013 | The clinical teacher | A Considerative Checklist to ensure safe daily patient review | Review: Narrative |
| Monash 2017 | Journal of hospital medicine | Standardized Attending Rounds to Improve the Patient Experience: A Pragmatic Cluster Randomized Controlled Trial | Wrong intervention |
| Mohta 2012 | BMJ quality & safety | The effects of a 'discharge time-out' on the quality of hospital discharge summaries | Wrong outcomes: discharge summaries |
| Morgan 2018 | The clinical teacher | Using simulation to prepare for clinical practice | Wrong outcomes: Students confidence |
| Mosher 2015 | BMC health services research | Aligning complex processes and electronic health record templates: A quality improvement intervention on inpatient interdisciplinary rounds Quality, performance, safety and outcomes | Wrong setting: IDR |
| Moussa 2019 | Journal of General Internal Medicine | Bedside rounds improve patient satisfaction and care transitions | Abstract / E- Poster |
| Murali-Krishnan 2019 | Journal of Endourology | Evaluation of effective urological ward rounds | Abstract / E - Poster |
| Nama 2016 | Anaesthesia and Intensive Care | Successful introduction of a daily checklist to enhance compliance with accepted standards of care in the medical intensive care unit | No intervention / comparator / baseline |
| Namboya 2017 | Infection | Daily goals for septic and non-septic patients in a tropical intensive care unit and causes for failure to achieve them | Abstract / E - Poster |
| Nassikas 2018 | Critical Care Medicine | ICU checklists keep cautis in check | Abstract / E - Poster |
| Newnham 2012 | Archives of Disease in Childhood | Impact of standardised documentation on post take ward round | Abstract / E - Poster |
| Noyes 2022 | British Journal of Surgery | Post-Take Pause Quality Improvement Project | Abstract / E - Poster |
| O'Brien 2015 | Critical Care Medicine | Critical care rounds: Standardizing key elements to ensure success | Abstract / E - Poster |
| O’Leary 2010 | Journal of hospital medicine | Improving patient safety: Impact of structured interdisciplinary rounds on a medical teaching unit | Abstract / E - Poster |
| O’Leary 2010 | Journal of hospital medicine | Improving teamwork: Impact of structured interdisciplinary rounds on a hospitalist unit | Wrong intervention |
| O'Leary 2011 | Archives of Internal Medicine | Structured interdisciplinary rounds in a medical teaching unit: improving patient safety | Wrong setting: non-bedside |
| O’Leary 2015 | American journal of medical quality : the official journal of the American College of Medical Quality | Implementation of unit-based interventions to improve teamwork and patient safety on a medical service | Wrong intervention |
| Olm-Shipman 2017 | Neurocritical care | Implementation of team-based communication strategies improves interprofessional collaboration in the neurosciences intensive care unit | Abstract / E - Poster |
| Olowo 2018 | Journal of hospital medicine | The resident is in charge! a novel approach to improve early hospital discharge rates | Abstract / E - Poster |
| Ortmayer 2013 | Pediatric Critical Care Medicine | The rounding tool: Nurse presentation in multidisplinary rounds | Abstract / E - Poster |
| Palin 2018 | Anaesthesia | Implementation of a simple ward round checklist to improve ICU care and aid multidisciplinary communication | Abstract / E - Poster |
| Patel 2014 | Chest | ICU rounding checklist implementation: Effect of accountability measures on physician compliance | Abstract / E - Poster |
| Patel 2017 | British Journal of Oral and Maxillofacial Surgery | Improving safety and efficacy of the Surgical Ward Round | Abstract / E - Poster |
| Patten 2013 | Internal Medicine Journal | Reducing length of stay in the AMU: A simple and sustainable approach | Abstract / E - Poster |
| Patton 2021 | British Journal of Surgery | Is the pen mightier than the scalpel? Improving the qualityand efficiency of Major Trauma ward rounds | Abstract / E - Poster |
| Pearson 2022 | Future healthcare journal | Using a checklist within simulation improves trainees' confidence on ward rounds | Compares simulation |
| Perkins 2022 | Pediatric Critical Care Medicine | ELECTRONIC NOTES: MAXIMISING EFFICIENCY AND PATIENT SAFETY | Abstract / E - Poster |
| Perry 2016 | Medsurg nursing : official journal of the Academy of Medical-Surgical Nurses | A Daily Goals Tool to Facilitate Indirect Nurse-Physician Communication During Morning Rounds on a Medical-Surgical Unit | Wrong outcomes |
| Phipps 2007 | Pediatric critical care medicine : a journal of the Society of Critical Care Medicine and the World Federation of Pediatric Intensive and Critical Care Societies | Assessment of parental presence during bedside pediatric intensive care unit rounds: effect on duration, teaching, and privacy | No intervention / comparator / baseline |
| Poole 2018 | Anaesthesia | ICU ward round checklist-a quality improvement project | Abstract / E - Poster |
| Prasher 2014 | International Journal of Surgery | Does a ward round checklist improve documentation and patient care? | Abstract / E - Poster |
| Pucher 2014 | The British journal of surgery | Randomized clinical trial of the impact of surgical ward-care checklists on postoperative care in a simulated environment | Compares simulation |
| Pucher 2014 | Journal of the American College of Surgeons | Surgical ward-care checklists improve postoperative care in a simulated environment: A randomized controlled trial | Compares simulation |
| Pucher 2013 | Journal of the American College of Surgeons | Ward simulation improves surgical ward round performance: A randomised controlled trial of a simulation-based curriculum | Abstract / E - Poster |
| Pucher 2014 | Annals of surgery | Ward simulation to improve surgical ward round performance: a randomized controlled trial of a simulation-based curriculum | Compares simulation |
| Qureishi 2012 | Clinical Otolaryngology | Improving the standards of medical documentation in an ENT ward | Abstract / E - Poster |
| Radia 2016 | Archives of Disease in Childhood | Diversifying documentation of the evening ward round | Abstract / E - Poster |
| Rai 2018 | European Urology, Supplements | Safer, faster, better: Improving the urology ward round & handover process (iWRAHP) | Abstract / E - Poster |
| Rama 2018 | International Journal of Surgery | Improving vascular surgical ward rounds through implementation of ward round checklists | Abstract / E - Poster |
| Ramjee 2016 | European Stroke Journal | Improving patient care utilising a standardised approach to ward rounds in an Hyper-Acute Stroke-Unit (HASU) | Abstract / E - Poster |
| Raval 2015 | Journal of medical systems | Development and implementation of an electronic health record generated surgical handoff and rounding tool | Wrong study design: investigates electronically created patient lists with hand written one |
| Redley 2020 | Internal Medicine Journal | Mixed methods quality evaluation of structured interprofessional medical ward rounds | Duplication |
| Rehder 2009 | Critical Care Medicine | Shared agreement of daily goals in the pediatric intensive care unit can be improved through targeted interventions | Abstract / E - Poster |
| Rej 2017 | Gut | Daily consultant delivered ward care: Effects on inpatient stay and patient care | Abstract / E - Poster |
| Remington 2018 | Paediatrics and Child Health (Canada) | Use of a daily rounding checklist on the clinical teaching unit improves interdisciplinary communication around discharge | Abstract / E - Poster |
| RenyElizabeth 2022 | Pediatric Hematology Oncology Journal | Incorporating structured nursing checklist in pediatric oncology day to day practice: A pilot experience | Abstract / E - Poster |
| Rice 2022 | Critical Care Medicine | Standardized interdisciplinary bedside rounding improves patient and family satisfaction | Abstract / E - Poster |
| Richardson 2017 | Stroke | Improving transitions of care with a daily rounding tool | Abstract / E - Poster |
| Roberts 2020 | British Journal of Surgery | A Multidisciplinary Approach: Utilising a Ward Round Proforma improves Patient Safety in a Tertiary Plastic Surgery Unit | Abstract / E - Poster |
| Robertson 2013 | International Journal of Stroke | A consultant delivered post take ward round (PTWR) checklist improves cardiopulmonary resuscitation (CPR) and ceiling of care (COC) decision-making on the hyperacute stroke unit (HASU) | Abstract / E - Poster |
| Ross 2022 | Age and Ageing | IPC STOCKINGS FOR VTE PREVENTION IN STROKE: BENEFITS OF INTRODUCING ELECTRONIC PRESCRIPTIONS ANDWARD ROUND PROMPTS | Abstract / E - Poster |
| Sammann 2020 | The Journal of surgical research | Improving Efficiency and Meeting Expectations Without Compromising Care on Trauma Surgical Rounds | Wrong study design: Interventions and aims are to move away from bedside |
| Schwartz 2018 | Journal of General Internal Medicine | Structured interdisciplinary bedside rounds improve the quality of inter-professional communication on an inpatient general medicine teaching unit | Wrong intervention |
| Schwartz 2021 | Journal of interprofessional care | Structured interdisciplinary bedside rounds improve interprofessional communication and workplace efficiency among residents and nurses on an inpatient internal medicine unit | Wrong intervention |
| Shabbir 2015 | BMJ quality improvement reports | Four Simple Ward Based Initiatives to Reduce Unnecessary In-Hospital Patient Stay: A Quality Improvement Project | Wrong intervention |
| Sharma 2021 | British Journal of Surgery | Does the introduction of ward round stickers improve surgical ward rounds? | Abstract / E - Poster |
| Sharp 2020 | Journal of General Internal Medicine | Bedside rounds observation: Patient centeredness of communication | Abstract / E - Poster |
| Shaughnessy 2015 | Nursing in critical care | Introduction of a new ward round approach in a cardiothoracic critical care unit | Wrong outcomes |
| Shields 2020 | Advances in medical education and practice | Is Asking Questions on Rounds a Teachable Skill? A Randomized Controlled Trial to Increase Attendings' Asking Questions | Wrong population: doctor outcomes of question asking |
| Siegel 2018 | Pediatric quality & safety | Impact of a Daily PICU Rounding Checklist on Urinary Catheter Utilization and Infection | No intervention / comparator / baseline |
| Sinvani 2017 | Journal of hospital medicine | Implementing ACOVE Quality Indicators as an Intervention Checklist to Improve Care for Hospitalized Older Adults | Wrong population |
| Smith 2018 | British Journal of Health Care Management | The impact of a ward round manager: A time and motion study | Wrong intervention |
| Somasundram 2018 | BJU international | Simulation in urology to train non-technical skills in ward rounds | Wrong outcomes: assessing NOTSS |
| Spoor 2015 | Archives of Disease in Childhood | Improving the standard of paediatric ward rounds | Abstract / E - Poster |
| Sproson 2017 | International Journal of Surgery | Implementing a urological ward round patient safety checklist: A complete audit cycle | Abstract / E - Poster |
| Steadman 2014 | Archives of Disease in Childhood | Electronic ward rounds and #handoverproject-improving quality while increasing efficiency | Abstract / E - Poster |
| Stein 2011 | Journal of hospital medicine | Combined effect of multidisciplinary bedside rounding and real-time visualization of prophylaxis status on hospital-acquired venous thromboembolism in a surgical intensive care unit | Abstract / E - Poster |
| Stickrath 2011 | Journal of General Internal Medicine | Interventions to improve attending rounds in medicine: A systematic review | Review |
| Stuhlreyer 2022 | BMC Medicine | A digital application and augmented physician rounds reduce postoperative pain and opioid consumption after primary total knee replacement (TKR): a randomized clinical trial | Wrong study design |
| Sturgess 2011 | Pediatric Critical Care Medicine | Reduction in prescription errors on paediatric intensive care with 'zero tolerance prescription' | Abstract / E - Poster |
| Tallent 2021 | Journal of the American Association of Nurse Practitioners | Extending the radius of family-centered care in the pediatric cardiac intensive care unit through virtual rounding | Wrong intervention |
| Tapper 2012 | Hepatology | A checklist as a quality improvement tool significantly reduces length of stay on an inpatient hepatology service | Abstract / E - Poster |
| TenHave 2013 | Critical Care | Leadership training and quality improvement of interdisciplinary rounds in the ICU | Abstract / E - Poster |
| TenHave 2013 | Intensive Care Medicine | Quality improvement of interdisciplinary rounds by leadership training based on essential quality indicators of the Interdisciplinary Rounds Assessment Scale | Wrong intervention |
| Thomas 2015 | BMJ quality & safety | Driven to distraction: a prospective controlled study of a simulated ward round experience to improve patient safety teaching for medical students | Compares simulation |
| Thomson 2011 | European Journal of Internal Medicine | Use of check-and-challenge for a medical ward-round checklist improves patient safety | Abstract / E - Poster |
| Tocci 2019 | Critical Care Medicine | The effect of an electronic decision support quality tool on intensive care unit rounds | Abstract / E - Poster |
| Townsend-Gervis 2014 | Western journal of nursing research | Interdisciplinary Rounds and Structured Communication Reduce Re-Admissions and Improve Some Patient Outcomes | Wrong population |
| Treloar 2022 | World journal of surgery | Can Checklists Solve Our Ward Round Woes? A Systematic Review | Review |
| Twohig 2020 | Journal of the Intensive Care Society | ICU microbiology ward round documentation | Abstract / E - Poster |
| Ullman 2011 | Pediatric Critical Care Medicine | Keeping 'kids safe': Facilitating best practice in the PICU | Abstract / E - Poster |
| Unaka 2019 | Hospital Pediatrics | Improving Efficiency of Pediatric Hospital Medicine Team Daily Workflow | Wrong outcomes |
| Ursprung 2005 | Quality & safety in health care | Real time patient safety audits: improving safety every day | Wrong study design |
| Uwaoma 2012 | Journal of hospital medicine | Effectiveness of patient centered team rounds at the bedside in an adult hospitalist practice | Abstract / E - Poster |
| Vats 2009 | Critical Care Medicine | A lean focused rounding process in a pediatric intensive care unit increases efficiency, optimizes resources, and improves satisfaction | Abstract / E - Poster |
| Vats 2011 | Pediatric critical care medicine : a journal of the Society of Critical Care Medicine and the World Federation of Pediatric Intensive and Critical Care Societies | Lean analysis of a pediatric intensive care unit physician group rounding process to identify inefficiencies and opportunities for improvement | Compares simulation |
| Vats 2012 | Critical Care Medicine | The impact of a lean rounding process in a pediatric intensive care unit | Wrong intervention |
| Waters 2012 | Intensive Care Medicine | Improving communication in a neuro-trauma critical care unit | Abstract / E - Poster |
| Watson 2015 | European Geriatric Medicine | Providing clear structure and leadership to an assessment area can reduce patient length of stay (LoS) and enhance staff experience | Abstract / E - Poster |
| Weiss 2011 | American Journal of Respiratory and Critical Care Medicine | Prompting to use a daily rounding checklist reduces costs associated with hospitalization | Abstract / E - Poster |
| Weiss 2010 | American Journal of Respiratory and Critical Care | Checklist-based prompting improves ICU outcomes | Abstract / E-Poster |
| White 2022 | British Journal of Surgery | Ward round documentation in vascular surgery-a closed loop audit cycle | Abstract / E - Poster |
| Wickersham 2016 | Journal of General Internal Medicine | Improving nurse-physician communication through a resident-led communication device | Abstract / E - Poster |
| Wickersham 2021 | American journal of medical quality : the official journal of the American College of Medical Quality | Making Room at the Bedside: Improving Communication Alongside Medical Education Through Interdisciplinary Rounds | Wrong outcomes |
| Woo 2015 | Academic Emergency Medicine | Interdisciplinary bedside rounds: Does a bedside, team-based approach improve patient perception of communication? | Abstract / E - Poster |
| Wu 2020 | Internal Medicine Journal | Marvelous ward rounds: A checklist method to general medical rounding | Abstract / E - Poster |
| Yates 2020 | Journal of the American Geriatrics Society | Implementation of age-friendly interdisciplinary rounds | Abstract / E - Poster |

**Appendix 5**

Appendix 5 – Hospital Length of stay meta-analysis. Forest plot comparing all studies reporting impact of checklist intervention on Hospital length of stay.

**
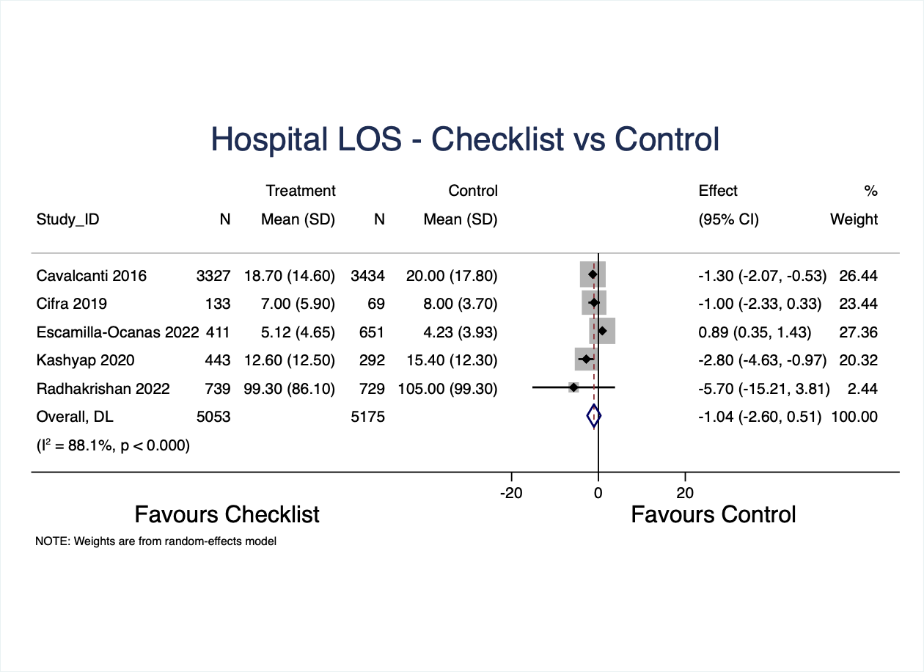
**


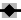
 **Effect size of individual study (SMD),
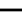
 95% confidence interval,**
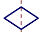
 **Combined effect estimate.**

**Appendix 6 -**

Appendix 6 – ICU mortality meta-analysis. Forest plot comparing all studies reporting impact of checklist intervention on ICU mortality.

**
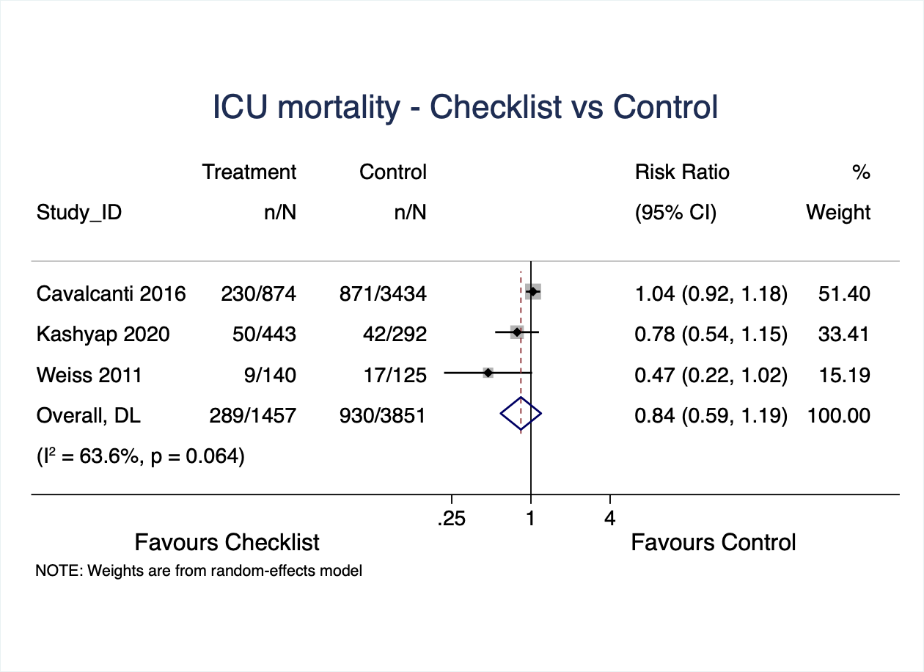
**


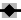
 **Effect size of individual study (SMD),
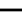
 95% confidence interval,**
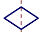
 **Combined effect estimate.**

**Appendix 7**

Appendix 7 – Inpatient mortality meta-analysis. Forest plot comparing all studies reporting impact of checklist intervention on Inpatient mortality.

**
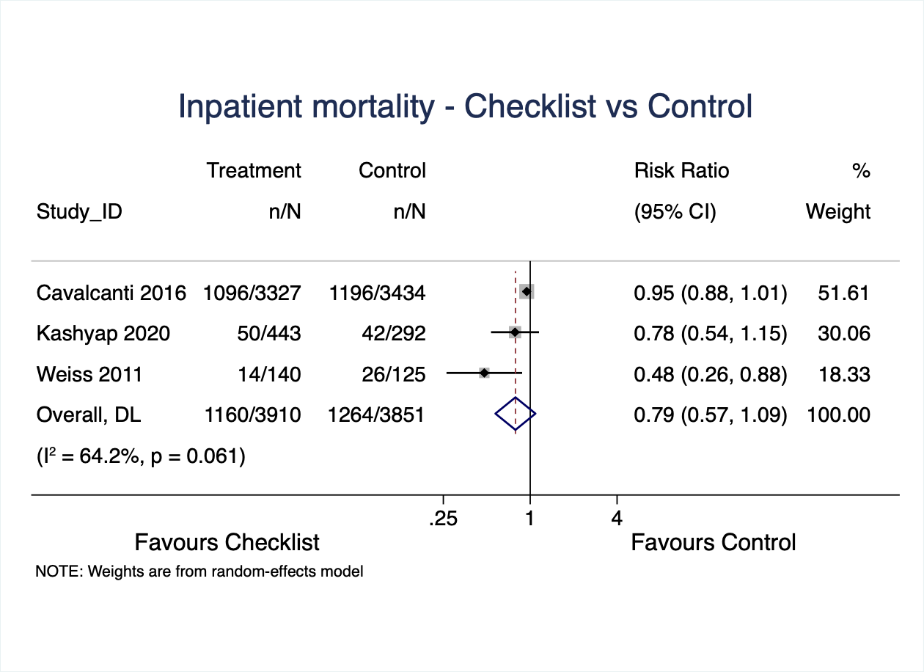
**


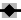
 **Effect size of individual study (SMD),
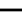
 95% confidence interval,**
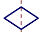
 **Combined effect estimate.**

**Appendix 8 – Significant Documentation**

Appendix 8a: Documentation of Observations meta-analysis. Forest plot comparing all studies reporting impact of checklist intervention on documentation of Observations.

**
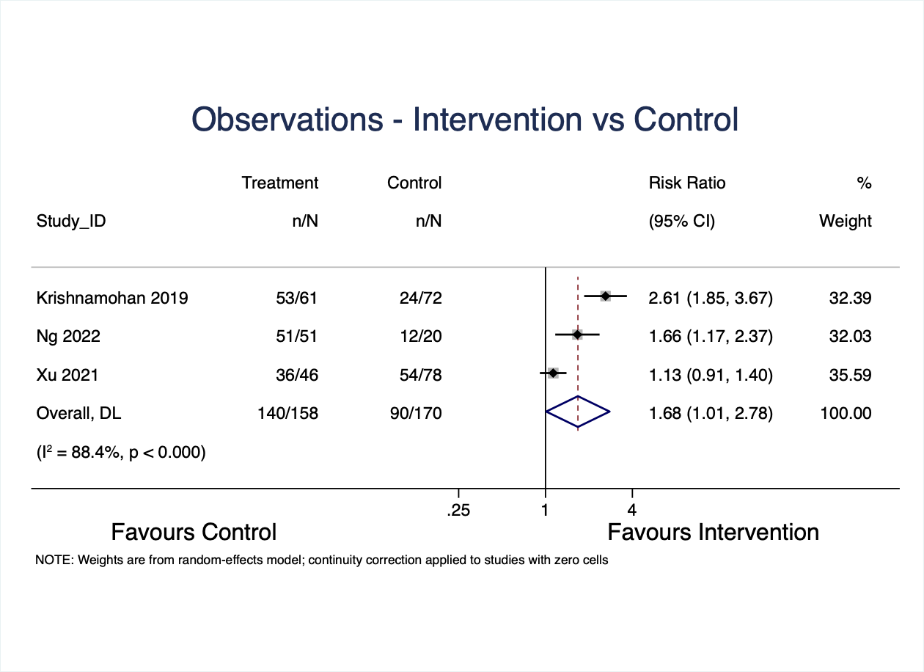
**

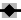
 **Effect size of individual study (SMD),
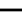
 95% confidence interval,**
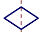
 **Combined effect estimate.**

Appendix 8b: Documentation of Diagnosis meta-analysis. Forest plot comparing all studies reporting impact of checklist intervention on documentation of Diagnosis.


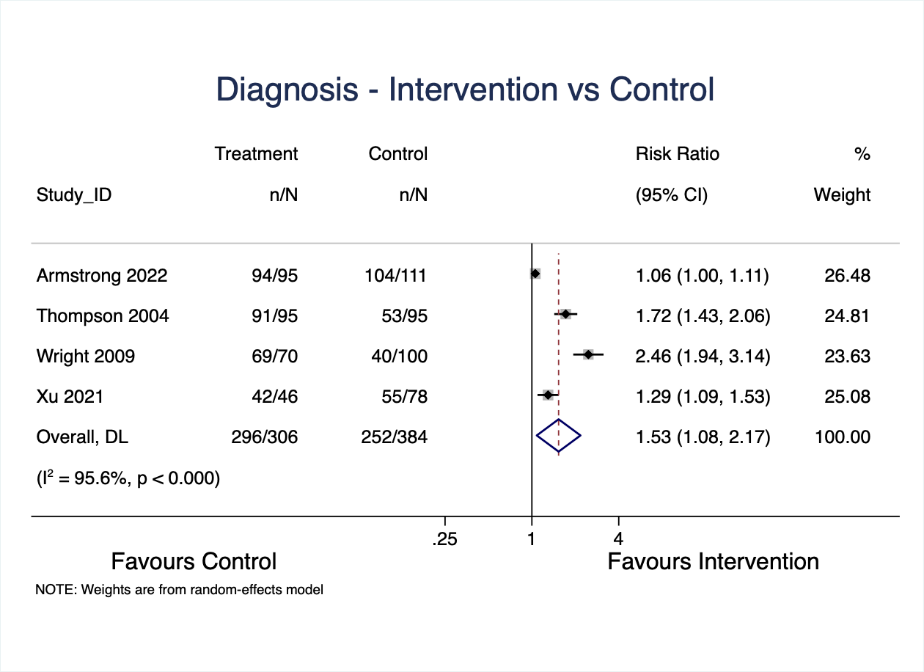


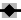
 **Effect size of individual study (SMD),
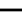
 95% confidence interval,**
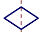
 **Combined effect estimate.**

Appendix 8c: Documentation of Impression meta-analysis. Forest plot comparing all studies reporting impact of checklist intervention on documentation of Impression.

**
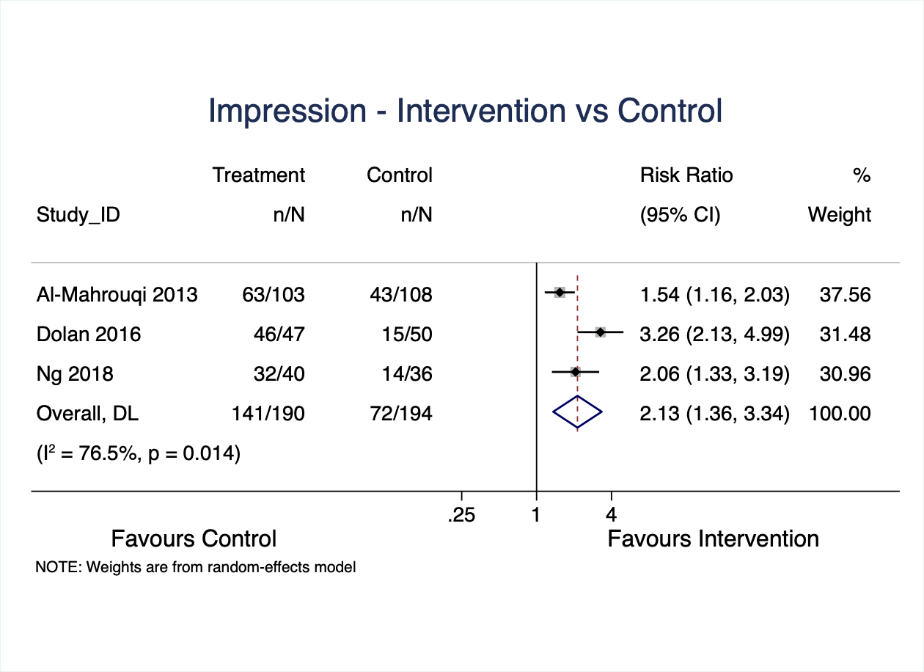
**

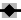
 **Effect size of individual study (SMD),
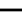
 95% confidence interval,**
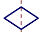
 **Combined effect estimate.**

Appendix 8d: Documentation of DVT meta-analysis. Forest plot comparing all studies reporting impact of checklist intervention on documentation of DVT.


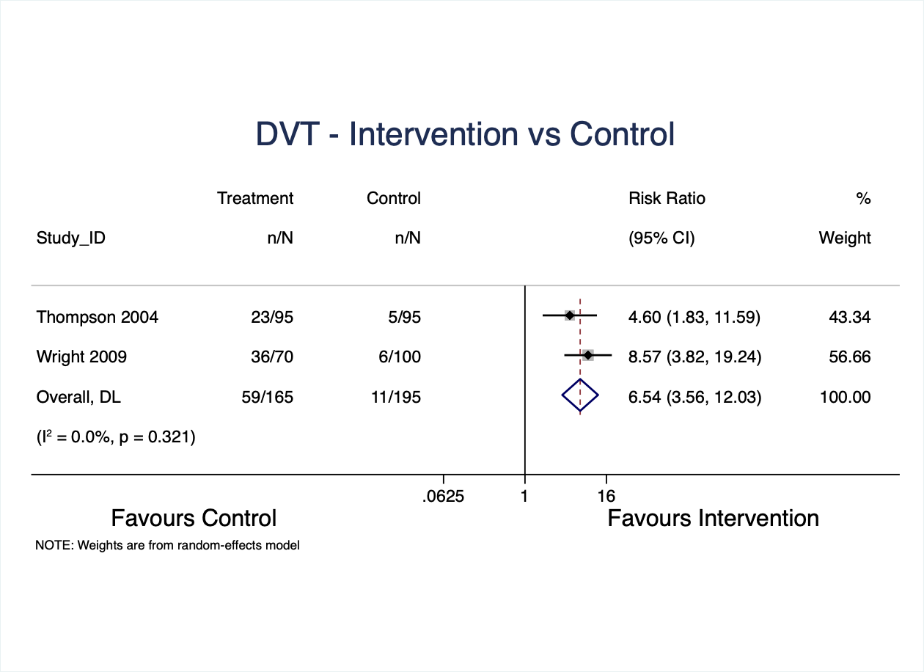


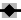
 **Effect size of individual study (SMD),
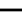
 95% confidence interval,**
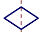
 **Combined effect estimate.**

Appendix 8e: Documentation of Resuscitation Status meta-analysis. Forest plot comparing all studies reporting impact of checklist intervention on documentation of Resuscitation Status.


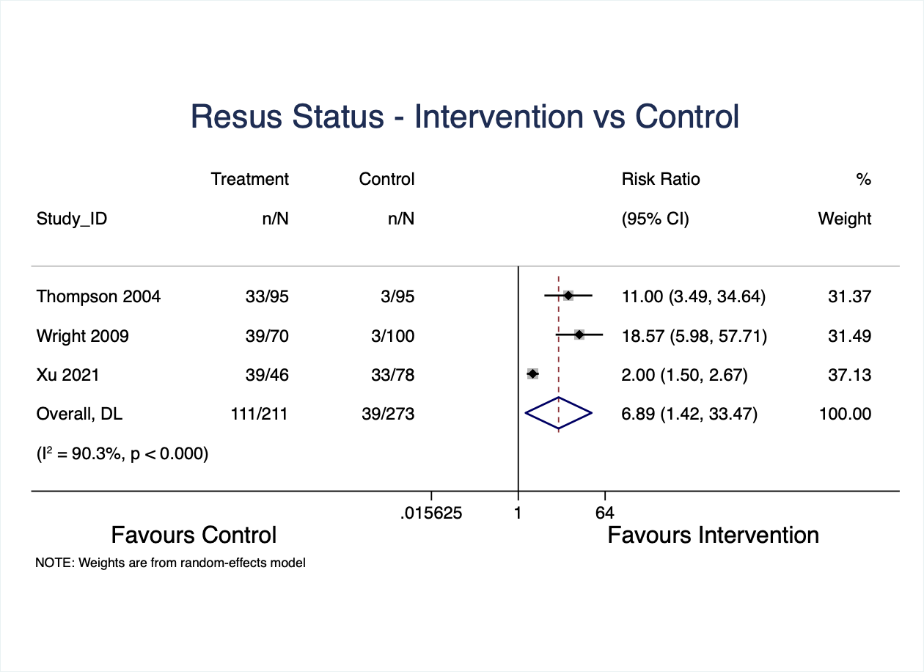


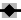
 **Effect size of individual study (SMD),
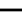
 95% confidence interval,**
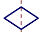
 **Combined effect estimate.**

Appendix 8f: Documentation of Drug Chart Review meta-analysis. Forest plot comparing all studies reporting impact of checklist intervention on documentation of Drug Chart Review

**
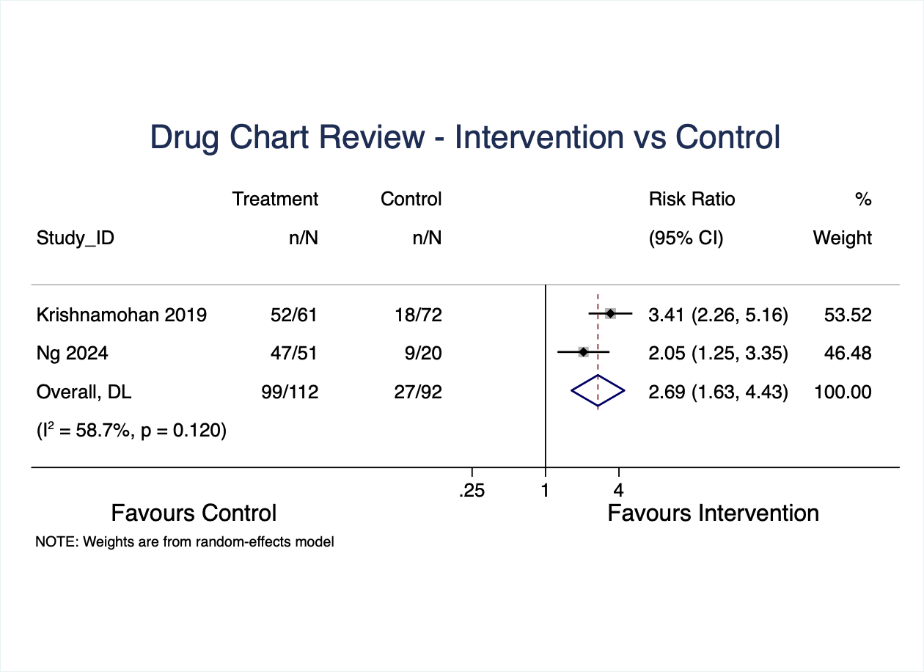
**
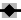
 **Effect size of individual study (SMD),
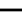
 95% confidence interval,**
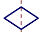
 **Combined effect estimate.**

Appendix 8g: Documentation of Bloods meta-analysis. Forest plot comparing all studies reporting impact of checklist intervention on documentation of Bloods.

**
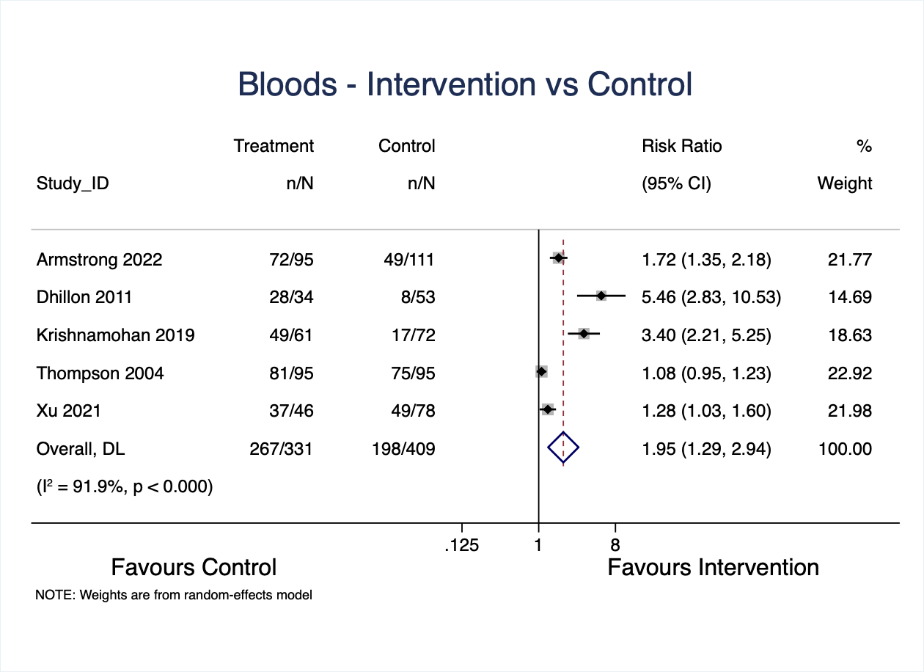
**
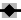
 **Effect size of individual study (SMD),
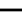
 95% confidence interval,**
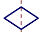
 **Combined effect estimate.**

Appendix 8h: Documentation of VTE meta-analysis. Forest plot comparing all studies reporting impact of checklist intervention on documentation of VTE

**
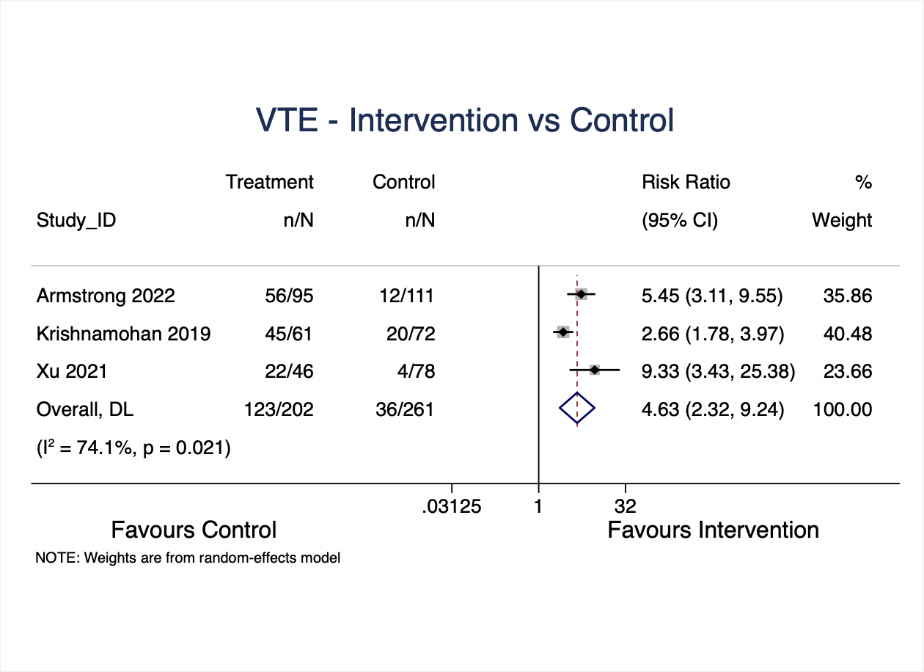
**
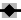
 **Effect size of individual study (SMD),
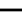
 95% confidence interval,**
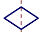
 **Combined effect estimate.**

Appendix 8i: Documentation of Bleep meta-analysis. Forest plot comparing all studies reporting impact of checklist intervention on documentation of Bleep.


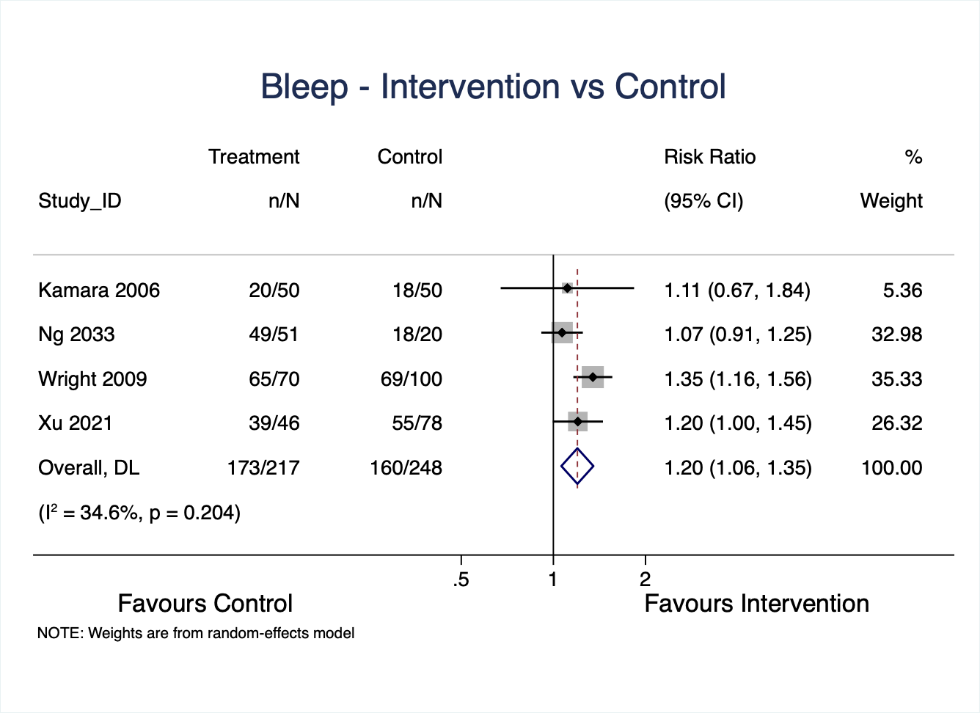


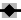
 **Effect size of individual study (SMD),
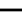
 95% confidence interval,**
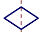
 **Combined effect estimate.**

Appendix 8j: Documentation of ECG meta-analysis. Forest plot comparing all studies reporting impact of checklist intervention on documentation of ECG.

**
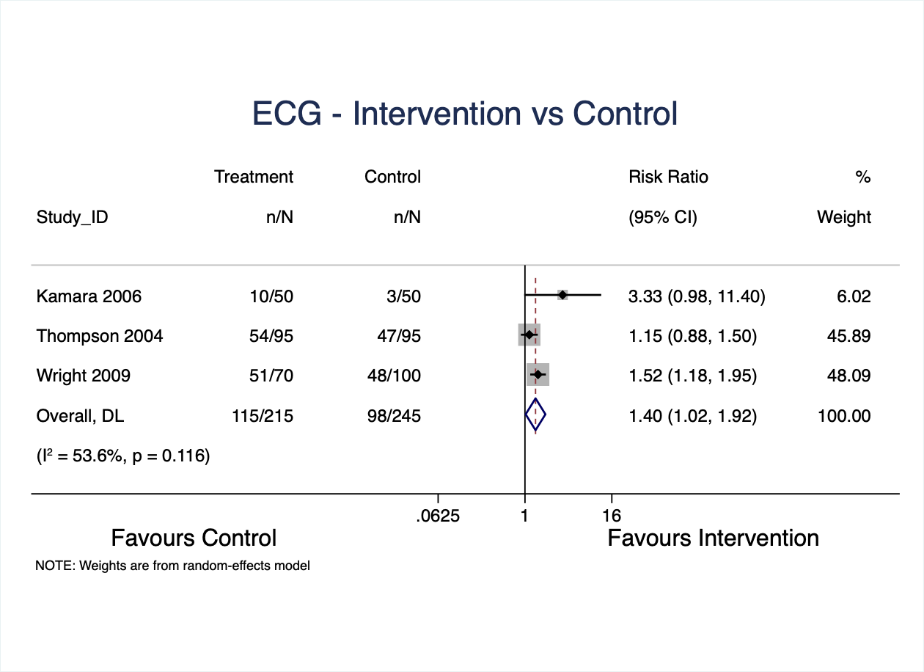
**
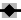
 **Effect size of individual study (SMD),
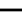
 95% confidence interval,**
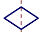
 **Combined effect estimate.**

Appendix 8k: Documentation of Plan meta-analysis. Forest plot comparing all studies reporting impact of checklist intervention on documentation of Plan.


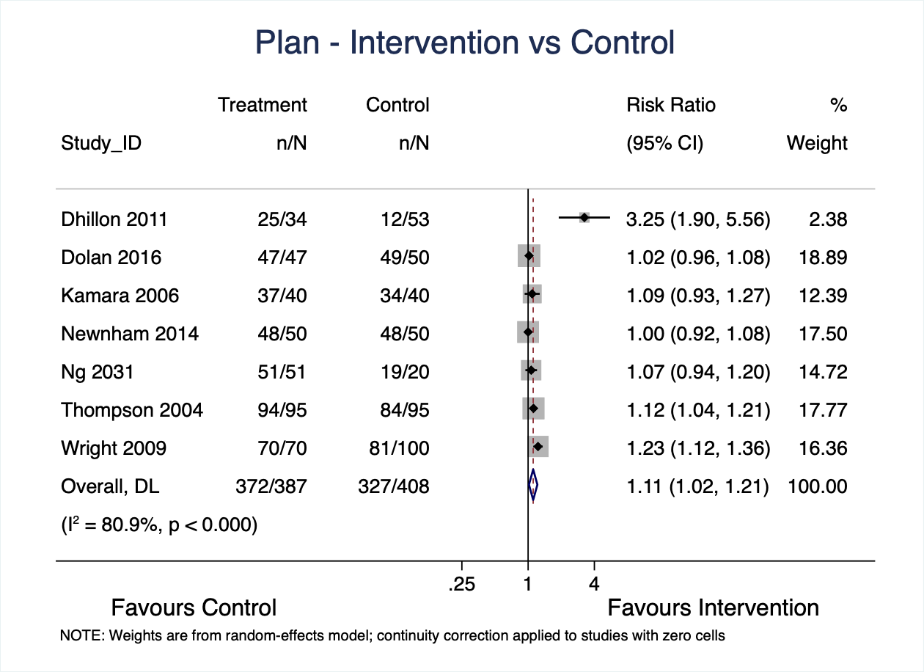


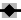
 **Effect size of individual study (SMD),
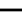
 95% confidence interval,**
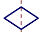
 **Combined effect estimate.**

Appendix 8l: Documentation of Discharge Planning meta-analysis. Forest plot comparing all studies reporting impact of checklist intervention on documentation of Discharge Planning.


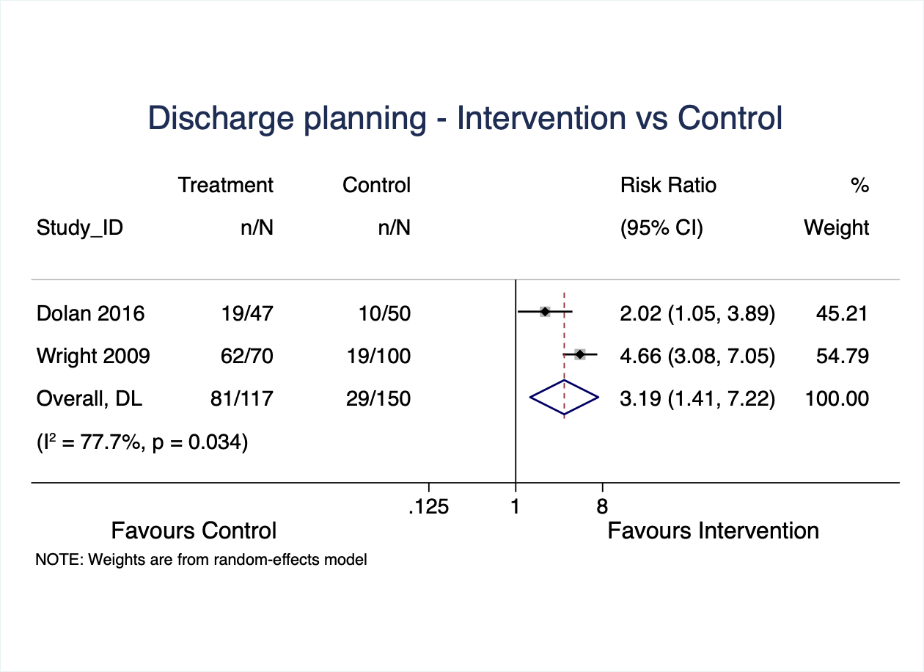

[
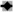
](https://www.thelancet.com/cms/10.1016/j.eclinm.2023.101893/asset/6989bb98-282a-47ca-8245-279e0be86bf8/main.assets/fx1_lrg.jpg) **Effect size of individual study (SMD),** [
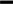
](https://www.thelancet.com/cms/10.1016/j.eclinm.2023.101893/asset/5abe3d28-44d6-48f2-b72d-58997b3760a7/main.assets/fx2_lrg.jpg) **95% confidence interval,**
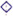
 **Combined effect estimate.**

**Appendix 9 – Non-significant Documentation**

Appendix 9a: Documentation of Lead of the Ward Round meta-analysis. Forest plot comparing all studies reporting impact of checklist intervention on documentation of Lead of the Ward Round.


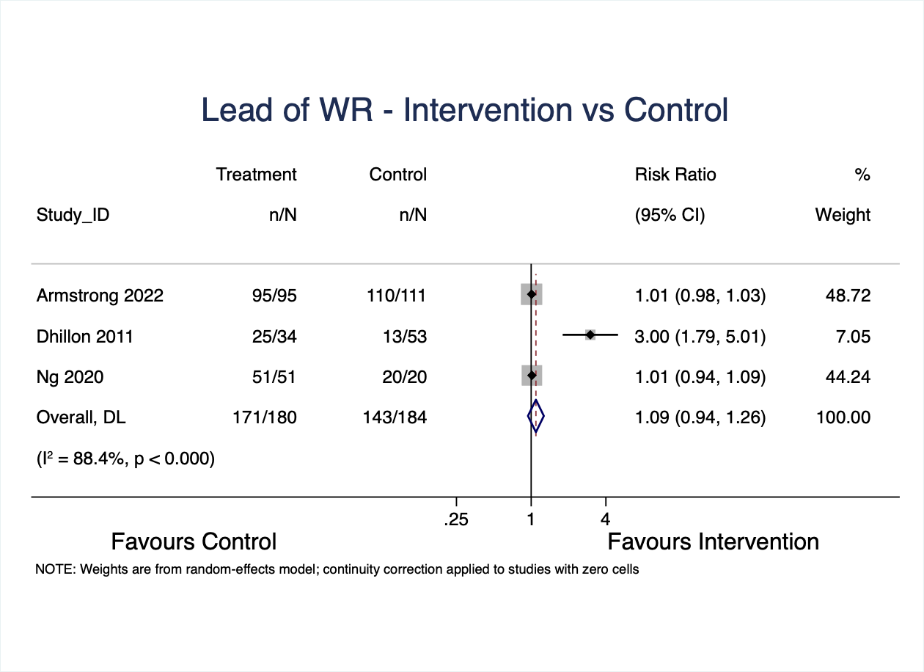


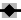
 **Effect size of individual study (SMD),
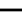
 95% confidence interval,**
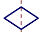
 **Combined effect estimate.**

Appendix 9b: Documentation of Time meta-analysis. Forest plot comparing all studies reporting impact of checklist intervention on documentation of Time.


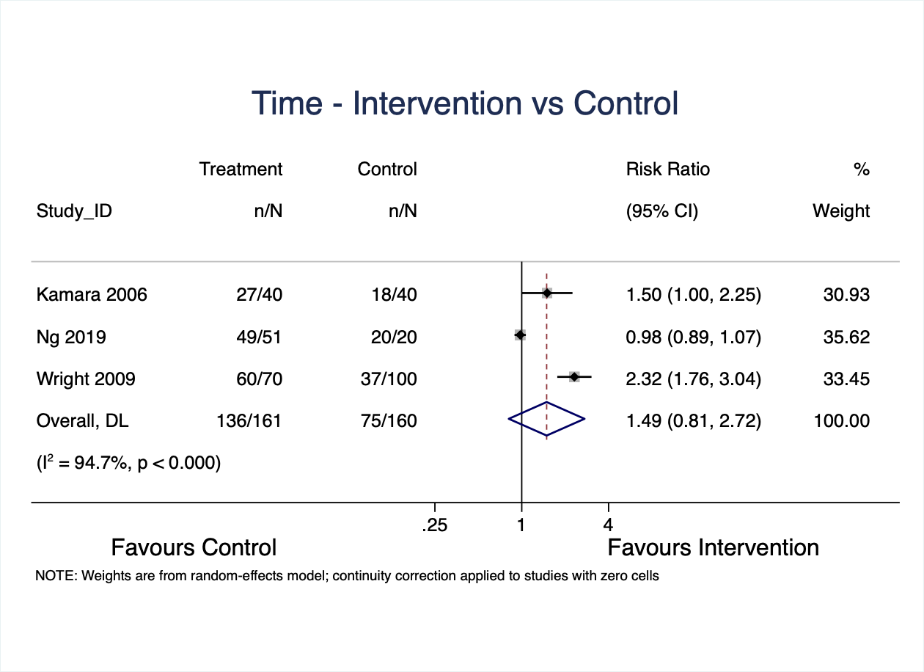


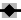
 **Effect size of individual study (SMD),
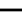
 95% confidence interval,**
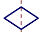
 **Combined effect estimate.**

Appendix 9c: Documentation of Signature meta-analysis. Forest plot comparing all studies reporting impact of checklist intervention on documentation of Signature.


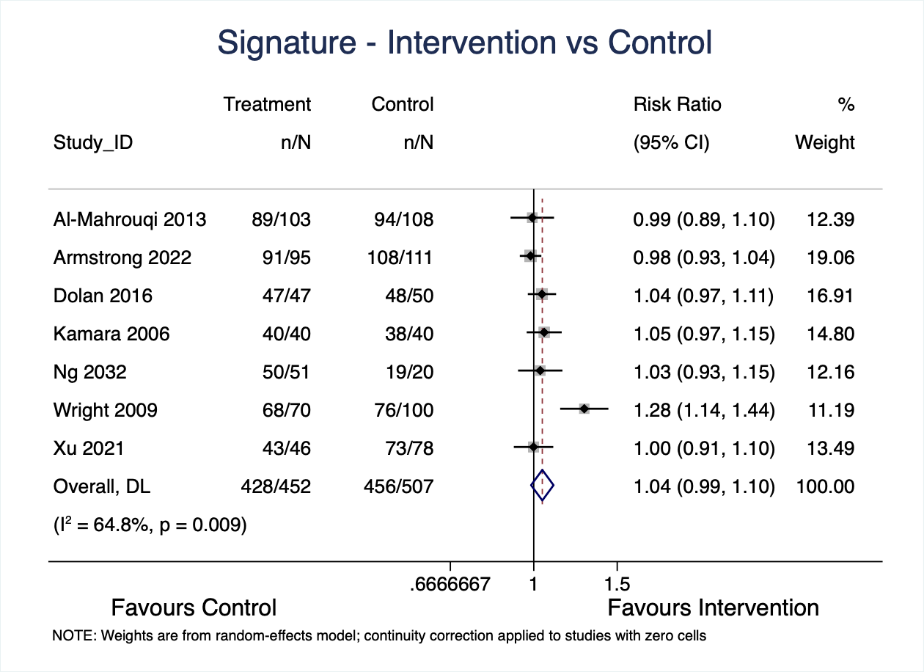


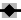
 **Effect size of individual study (SMD),
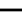
 95% confidence interval,**
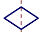
 **Combined effect estimate.**

Appendix 9d: Documentation of Date meta-analysis. Forest plot comparing all studies reporting impact of checklist intervention on documentation of Date.


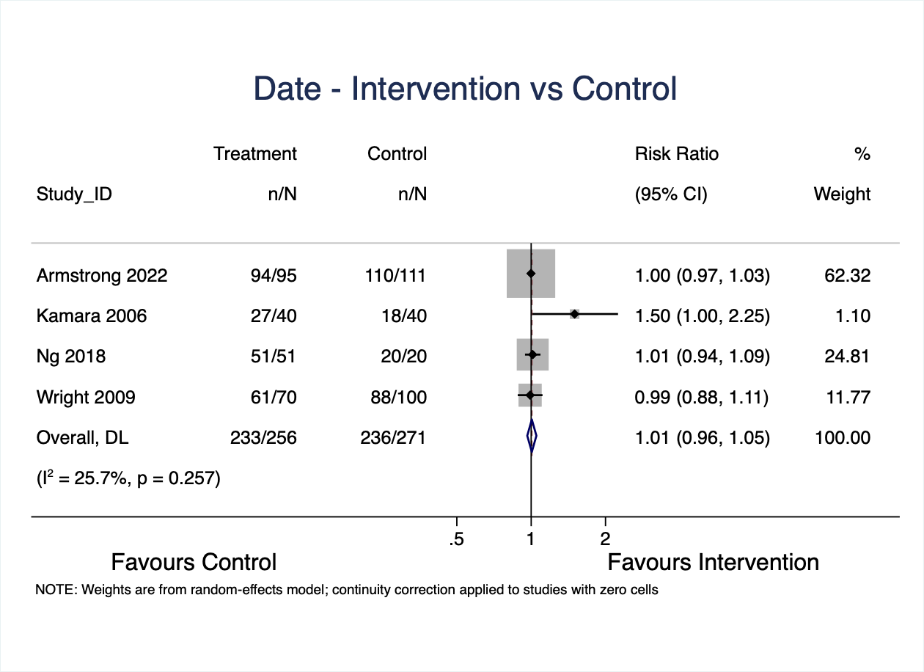


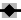
 **Effect size of individual study (SMD),
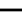
 95% confidence interval,**
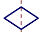
 **Combined effect estimate.**

Appendix 9e: Documentation of Dietary plan meta-analysis. Forest plot comparing all studies reporting impact of checklist intervention on documentation of Dietary plan.

**
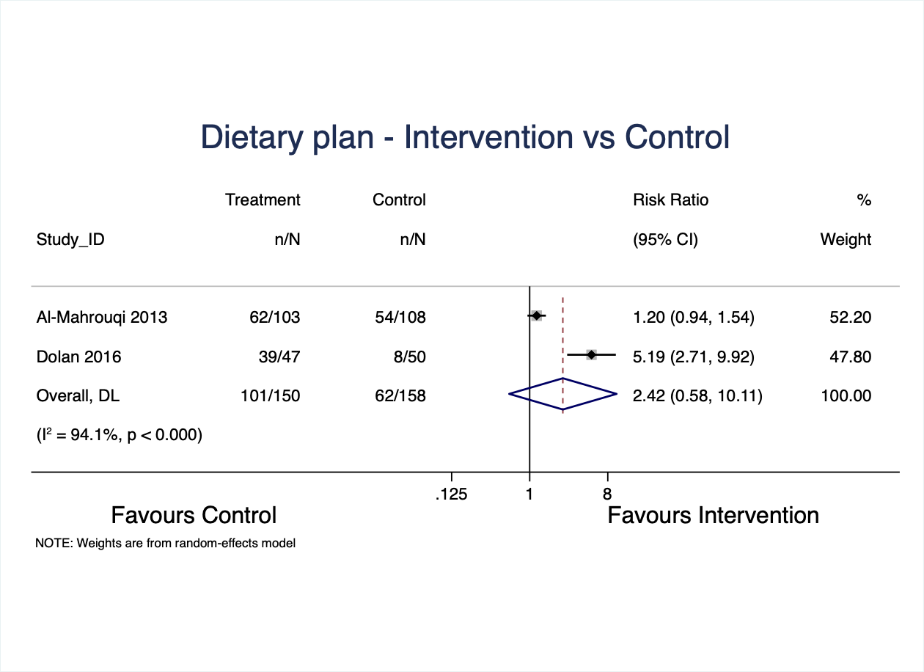
**
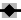
 **Effect size of individual study (SMD),
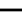
 95% confidence interval,**
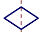
 **Combined effect estimate.**

Appendix 9f: Documentation of Hospital number meta-analysis. Forest plot comparing all studies reporting impact of checklist intervention on documentation of Hospital number.

**
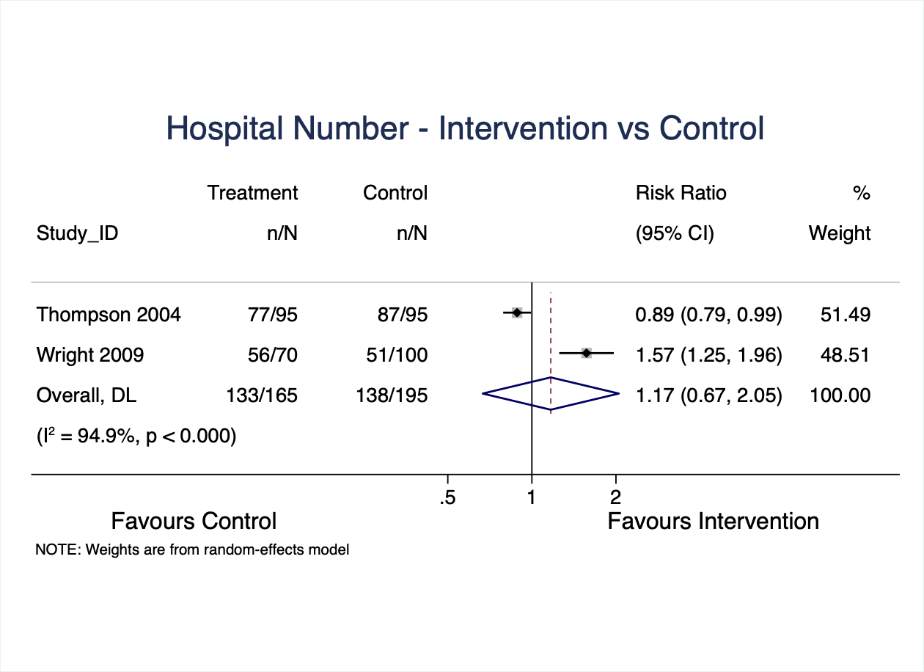
**


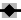
 **Effect size of individual study (SMD),
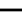
 95% confidence interval,**
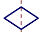
 **Combined effect estimate.**

Appendix 9g: Documentation of Examination meta-analysis. Forest plot comparing all studies reporting impact of checklist intervention documentation of Examination.


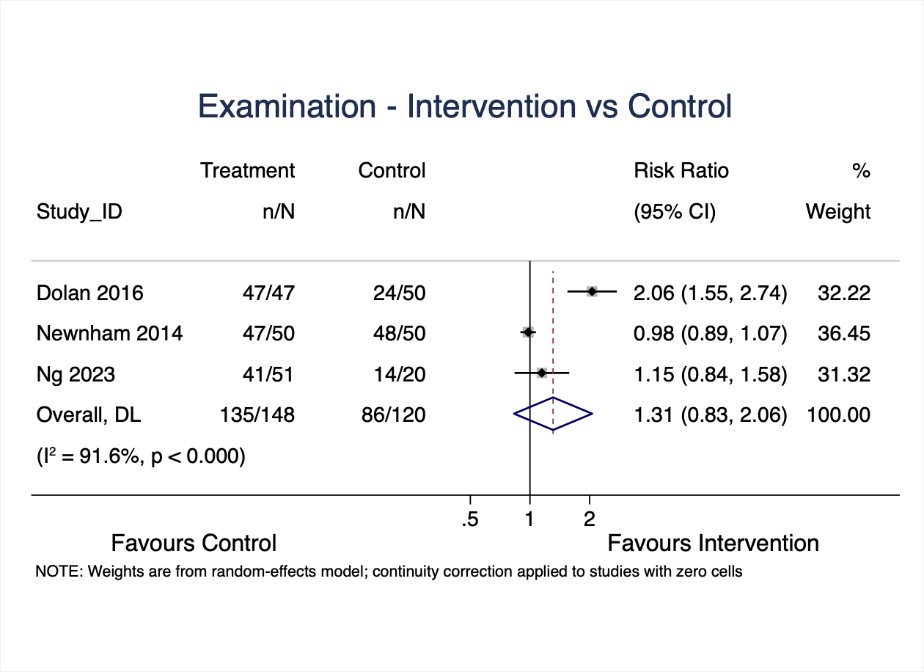


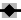
 **Effect size of individual study (SMD),
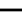
 95% confidence interval,**
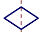
 **Combined effect estimate.**

Appendix 9h: Documentation of Consultant Name stay meta-analysis. Forest plot comparing all studies reporting impact of checklist intervention on documentation of Consultant Name.


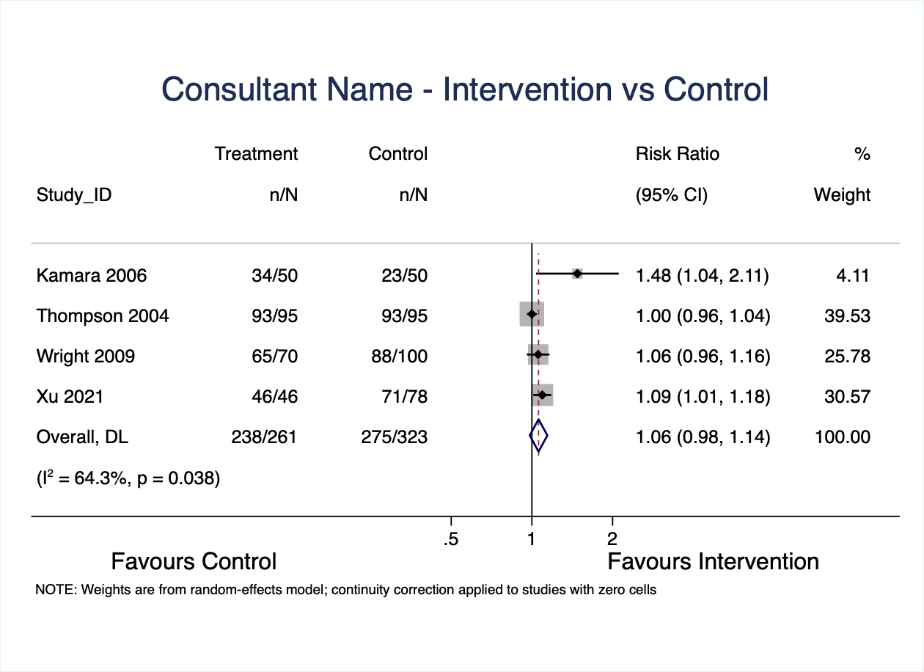


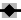
 **Effect size of individual study (SMD),
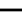
 95% confidence interval,**
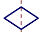
 **Combined effect estimate.**

Appendix 9i: Documentation of CXR meta-analysis. Forest plot comparing all studies reporting impact of checklist intervention on documentation of CXR.


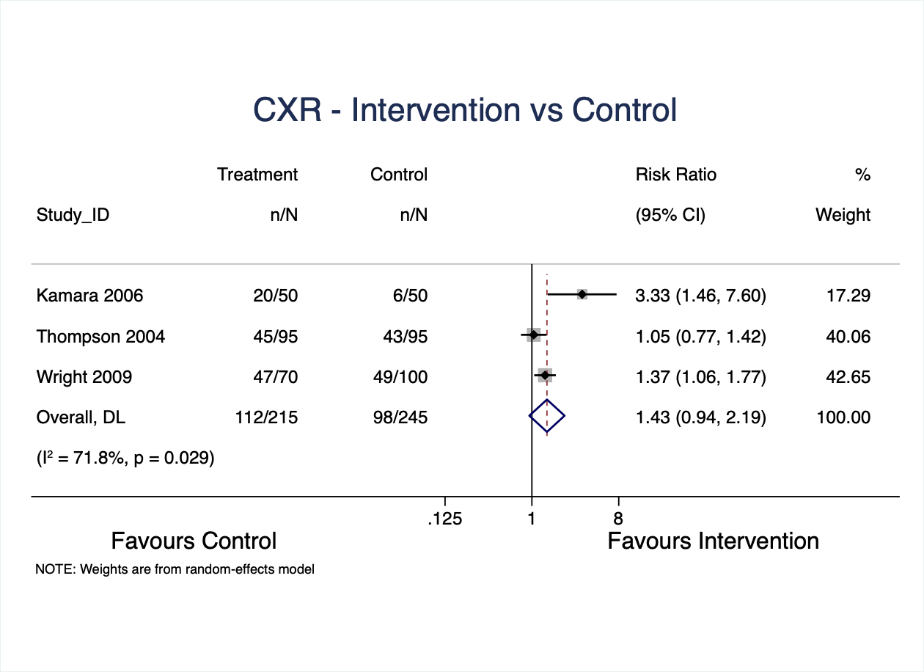


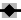
 **Effect size of individual study (SMD),
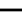
 95% confidence interval,**
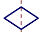
 **Combined effect estimate.**

Appendix 9j: Documentation of Grade meta-analysis. Forest plot comparing all studies reporting impact of checklist intervention on documentation of Grade.

**
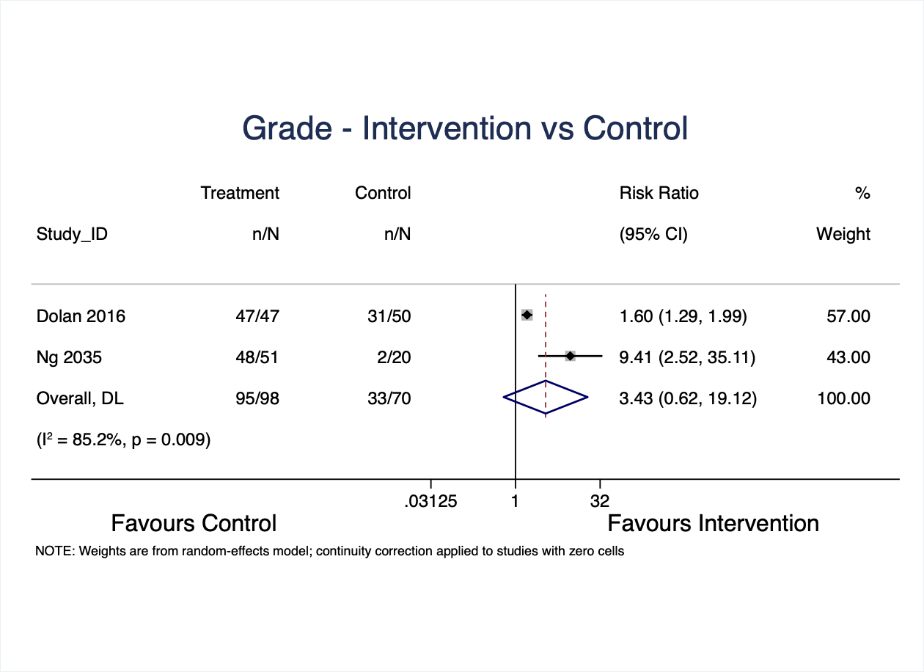
**
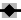
 **Effect size of individual study (SMD),
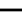
 95% confidence interval,**
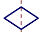
 **Combined effect estimate.**

Appendix 9k: Documentation of Patient name meta-analysis. Forest plot comparing all studies reporting impact of checklist intervention on documentation of Patient name.

**Effect size of individual study (SMD), 95% confidence interval,**  **Combined effect estimate.**

Appendix 9l: Documentation of Vitals meta-analysis. Forest plot comparing all studies reporting impact of checklist intervention on documentation of Vitals.

**Effect size of individual study (SMD), 95% confidence interval,**  **Combined effect estimate.**

**Appendix 10 – Checklist Intervention and Qualitative Outcomes Summary**

| Patient outcome | Study | Results | Sample Size |
| --- | --- | --- | --- |
| VTE | Johnston 2022 | "VTE prescribing and documentation improved by a rate ratio 1.25 (95% CI: 1.04 to 1.50)" | 189 |
| Antibiotics | Johnston 2022 | "Antibiotics prescribing improved by 1.44 (95% CI: 1.06 to 1.94)" | 189 |
| UTI | Escamilla-Ocañas 2022 | "Reduced urinary tract infections (UTIs) (OR 0.23, 95% CI: 0.09 to 0.55)” | 1062 |
| Infection | Escamilla-Ocañas 2022 | "Reduced rate of any new infections (OR 0.59, 95% CI: 0.40 to 0.87)" | 1062 |
|  | Radhakrishnan 2022 | "Faithful implementation eliminated central line-associated bloodstream infections and catheter-associated urinary tract infections" | Not reported |
| Duration of IV fluids and lines | Clark 2019 | "No clinically significant change was appreciated for IV medication duration" | 2707 |
| Adverse Events / clinical errors | Krishnamohan 2019 | **Reported adverse events (No.)** Prescription errors: 13 > 2 Antibiotic errors: 4 > 0 Fluid Balance Errors: 2 > 0  Patient observation chart errors: 2 > 0  VTE cases Diagnosed: 11 > 10 | 133 |
|  | Lepee 2012 | “After introduction of Check and Correct, there was a significant drop of adverse events −5.0 % (−37.7 % relative decrease; p<0.0001)” | 227 |
| Family satisfaction | Trahan | Important information discussed: P = 0.010 Understood the plan: P=0.002 Understand timing of discharge: P < 0.001 Felt included: P = 0.002 | 59 |
|  | Glick 2022 | Discussion of HAC risk factors: 11% > 89% Discussion of discharge planning: 60% > 92% Asking families for questions: 61% > 87% | 2447 |

**Appendix 11 –**

Appendix 11 – Time spent rounding per patient meta-analysis. Forest plot comparing all studies reporting impact of structure intervention on time spent rounding per patient.

**Effect size of individual study (SMD), 95% confidence interval,**  **Combined effect estimate.**

**Appendix 12 –**

Appendix 12 – Time spent rounding overall meta-analysis. Forest plot comparing all studies reporting impact of structure intervention on time spent rounding overall.

**Effect size of individual study (SMD), 95% confidence interval,**  **Combined effect estimate.**

**Appendix 13 –**

Appendix 13 – 30-day readmission meta-analysis. Forest plot comparing all studies reporting impact of structure intervention on 30-day readmission.

**Effect size of individual study (SMD), 95% confidence interval,**  **Combined effect estimate.**

**Appendix 14 –**

Appendix 14 – Length of stay meta-analysis. Forest plot comparing all studies reporting impact of structure intervention on Length of stay.

**Effect size of individual study (SMD), 95% confidence interval,**  **Combined effect estimate.**

**Appendix 15 – Structure intervention Qualitative Outcomes Summary**

| Patient outcome | Study | Results | Sample Size |
| --- | --- | --- | --- |
| Rates of falls | Urisman 2018 | "The rate of falls decreased from 9 to 0 per 1000 ICU patient-days. However, due to the rarity of these events, did not reach statistical significance" | 169 |
| Length of Stay | AcalJimenez 2018 | "The length of stay decreased from 2.5 days prior to the study, to 2.03 days during the first 4 weeks of the project, to 2.18 days during the remaining 4 weeks respectively" | 213 |
| Teaching | Cao 2016 | "There was greater teaching provided by the attending physician on the intervention team, with at least one teaching point provided on 51.2% of patient encounters versus 33.9% on the control team (p < 0.01)." | 665 |
| Hand hygiene | Christensen 2017 | "Improved adherence to adequate hand hygiene was also observed; it was recorded for 38 (54%) rounds before standardisation and during 65 (82%) rounds after the introduction of the structured ward round (P<0.001)." | 150 |
| Discharge Time | Shirreff 2019 | "Mean discharge time was earlier in the ± intervention phase 11:18 am 1 hour 59 minutes vs. baseline phase 12:37 PM ± 2 hours 37 minutes, P ˂ 0.001) with the rate of discharge before 11:00 AM almost doubling (post-intervention 69% vs. baseline 36%, P < 0.001, 95% CI: 19%−45%). | 223 |
| Post-operative morbidity | Ellison 2007 | "There were no differences in the observed rates of overall, major, or minor morbidities between the 2 study arms" | 270 |

**Appendix 16 – ‘Other’ intervention types and Qualitative Outcomes Summary**

| Study | Intervention | Sample Size | Outcomes | Results |
| --- | --- | --- | --- | --- |
| Spaner 2017 | Education | 21 | Documentation of ‘Goals of Care’ | April 2014: 3/14 (21%) 🡪 March 2015: 14/15 (93%) |
| Lienard 2010 | Education | 88 | Patient satisfaction | Patients’ satisfaction levels were higher in the training group (Median = 92; Q1–Q3 = 87–97) compared to the waiting-list group (Median = 88, Q1–Q3 = 83–95 |
| Harman 2019 | Education | 527 | Shared decision making (RPAD) | RPAD score improvement was 1.68 points (95% CI, 1.33–2.03; P < .001; Cohen d = 0.82) |
| Eden 2022 | Education | 160 | 1. Change in management. 2. Hospital 7 and 30- day readmission | 1.Change in management  “The intervention led to a change in management in 18% of observed patient encounters”.  2. Hospital readmission  “Not statistically different at 7 (10.1% vs 9.6%, P = 0.611) or 30 days (21% vs 22%, P = 0.466) |
| Torregrosa 2016 | Traffic Light System | N/A | Bed turnover rate | “5.8 to 6.3 in 12 months” |
| Roberts 2016 | Early vs Late Rounding | 152 | 1. Patient satisfaction scores  2. Discharge time | 1. Patient satisfaction  “Satisfaction was higher in the delayed compared with the early rounding group (median 9.0 [interquartile range 7.0–9.0] compared with 7.0 [6.0–8.0]; P,.01”  2.Discharge time:  No differences in discharge time |
| Murphy 2015 | Patient Information Sheet | 200 | Patient feedback | “76% knew their treatment plan for the day versus 41% (p<0.01)” |
| Southwick 2014 | Application of athletic principles | 780 | 1. Duration of round 2. Length of stay 3. 30-day readmission | 1. Duration of the round “The mean duration of rounds ± standard error of the mean (SEM) for the experimental group (110.8 ± 2.8, n = 75) was 16 minutes shorter than for the control group (126.1 ± 3.8, n = 64) (P = .0049).” 2. Length of stay Not statistically different 3. 30-day readmission 30% fewer discharges within 30 days in both phases of the trial (mean readmission percentage ± SEM: experimental group: 6.95±1.29, n = 576 discharges versus control group: 9.95±1.02, n = 659 discharges; P = .039) |
| Byrd 2018 | Mobile Devices | 76 | Time spent on round | “The average number of minutes spent per patient decreased after integrating the mobile devices from 11.5 to 9.6 minutes” |

**Supplementary Appendixes**

**Appendix 17– Risk of Bias Assessment for observational studies (Newcastle Ottawa – Scale)**

| **Author, Year** | **Very High (0-3)** | **High (4-6)** | **Moderate (7)** | **Low (8-9)** |
| --- | --- | --- | --- | --- |
| Abraham 2019 |  | * |  |  |
| AcalJimenez 2018 | * |  |  |  |
| Banfield 2017 | * |  |  |  |
| Alazzawi 2016 | * |  |  |  |
| Al-Mahrouqi 2013 |  | * |  |  |
| Armstrong 2022 |  | * |  |  |
| Blucher 2013 | * |  |  |  |
| Boland 2015 |  | * |  |  |
| Brown 2021 |  | * |  |  |
| Brown 2019 | * |  |  |  |
| Byrd 2018 |  | * |  |  |
| Cao 2017 |  | * |  |  |
| Chow 2019 |  | * |  |  |
| Christensen 2017 |  | * |  |  |
| Christensen 2022 |  | * |  |  |
| Cifra 2019 |  | * |  |  |
| Clark 2019 |  | * |  |  |
| Conroy 2015 |  |  | * |  |
| Crowson 2016 | * |  |  |  |
| De Bie 2021 |  |  | * |  |
| Dhillon 2011 |  | * |  |  |
| Dodek 2003 |  | * |  |  |
| Dolan 2016 |  | * |  |  |
| Duxbury 2013 | * |  |  |  |
| Eden 2022 | * |  |  |  |
| Efune 2018 |  | * |  |  |
| Escamilla-Ocanas 2022 |  |  | * |  |
| Feinman 2022 | * |  |  |  |
| Fleischmann 2015 |  | * |  |  |
| Galloway 2022 | * |  |  |  |
| Gilliland 2018 | * |  |  |  |
| Glick 2022 |  | * |  |  |
| Hale 2015 | * |  |  |  |
| Harmon 2019 |  | * |  |  |
| Johnston 2022 |  | * |  |  |
| Justice 2016 | * |  |  |  |
| Kamara 2006 | * |  |  |  |
| Kashyap 2020 |  | * |  |  |
| Keller 2018 |  | * |  |  |
| Khan 2018 |  | * |  |  |
| Koumoullis 2020 | * |  |  |  |
| Krishnamohan 2019 |  | * |  |  |
| Lepee 2012 |  | * |  |  |
| Licata 2013 | * |  |  |  |
| Ludley 2023 | * |  |  |  |
| Murphy |  | * |  |  |
| Nassikas 2020 |  | * |  |  |
| Newnham 2014 |  | * |  |  |
| Ng 2018 | * |  |  |  |
| Palmer 2013 |  | * |  |  |
| Parwaiz 2022 |  | * |  |  |
| Pitcher 2015 |  | * |  |  |
| Radhakrishnan 2022 | * |  |  |  |
| Redly 2019 |  | * |  |  |
| Rehder 2012 | * |  |  |  |
| Sharma 2013 |  | * |  |  |
| Shirreff 2019 |  | * |  |  |
| Simon 2020 |  | * |  |  |
| Southwick 2014 | * |  |  |  |
| Spaner 2017 | * |  |  |  |
| Stroud 2012 |  | * |  |  |
| Sunkara 2019 |  | * |  |  |
| Talia 2017 |  | * |  |  |
| Thompson 2003 | * |  |  |  |
| Trahan 2022 | * |  |  |  |
| Tranter-Entwistle 2020 |  | * |  |  |
| Urisman 2017 | * |  |  |  |
| Torregrossa | * |  |  |  |
| Vukanic 2021 |  | * |  |  |
| Weiss 2011 |  | * |  |  |
| Wright 2009 |  | * |  |  |
| Xu 2021 | * |  |  |  |

**Appendix 18 - Risk of bias assessment. 12 RCT studies were evaluated for risk of bias using the Cochrane Handbook RoB2 tool.**

| **Study ID** | **D1** | **D2** | **D3** | **D4** | **D5** | **Overall** |
| --- | --- | --- | --- | --- | --- | --- |
| Becker 2021 |  |  |  |  |  |  |
| Cavalcanti 2016 |  |  |  |  |  |  |
| Clarke-Pounder 2015 |  |  |  |  |  |  |
| Cox 2017 |  |  |  |  |  |  |
| Donovan 2020 |  |  |  |  |  |  |
| Ellison 2007 |  |  |  |  |  |  |
| Finn 2018 |  |  |  |  |  |  |
| Jaberi 2020 |  |  |  |  |  |  |
| Lienard 2010 |  |  |  |  |  |  |
| Osborn 2021 |  |  |  |  |  |  |
| Roberts 2016 |  |  |  |  |  |  |
| Read 2021 |  |  |  |  |  |  |

| \|  \|  \| \| --- \| --- \| | | Low risk |  |  |
| --- | --- | --- | --- | --- | --- | --- |
| \|  \| \| --- \| | | Some concerns |  |  |
| \|  \| \| --- \| | | High risk |  |  |
|  | |  |  |  |
| **D1** | **Randomisation process** | | |  |
| **D2** | **Deviations from the intended interventions** | | |  |
| **D3** | **Missing outcome data** | | |  |
| **D4** | **Measurement of the outcome** | | |  |
| **D5** | **Selection of the reported result** | | |  |

**References**

1. Johnson H, O'Farrell A, McKeown D, Sayers G, Hayes C, Beaton D. Is Increasing Life Expectancy Leading To More Complexity? Ir Med J. 2018;111(1):672.

2. Committee on the Learning Health Care System in A, Institute of M. In: Smith M, Saunders R, Stuckhardt L, McGinnis JM, editors. Best Care at Lower Cost: The Path to Continuously Learning Health Care in America. Washington (DC): National Academies Press (US)

Copyright 2013 by the National Academy of Sciences. All rights reserved.; 2013.

3. Nikendei C, Kraus B, Schrauth M, Briem S, Junger J. Ward rounds: how prepared are future doctors? Med Teach. 2008;30(1):88-91.

4. Cohn A. The ward round: what it is and what it can be. Br J Hosp Med (Lond). 2014;75 Suppl 6:C82-85.

5. Pucher PH, Aggarwal R, Srisatkunam T, Darzi A. Validation of the simulated ward environment for assessment of ward-based surgical care. Ann Surg. 2014;259(2):215-221.

6. Ghaferi AA, Birkmeyer JD, Dimick JB. Variation in hospital mortality associated with inpatient surgery. N Engl J Med. 2009;361(14):1368-1375.

7. Royal College of Physicians RCoN. Ward Rounds in Medicine: Principles for Best Practice. RCP, London; 2012.

8. GMC. Good Medical Practice. Manchester: General Medical Council; 2013.

9. Krishnamohan N, Maitra I, Shetty VD. The surgical ward round checklist: Improving patient safety and clinical documentation. J Multidiscip Healthc. 2019;12:789-794.

10. Zegers M, de Bruijne MC, Spreeuwenberg P, Wagner C, Groenewegen PP, van der Wal G. Quality of patient record keeping: an indicator of the quality of care? BMJ Qual Saf. 2011;20(4):314-318.

11. Shetty K, Poo SXW, Sriskandarajah K, Sideris M, Malietzis G, Darzi A, et al. "The Longest Way Round Is The Shortest Way Home": An Overhaul of Surgical Ward Rounds. World J Surg. 2018;42(4):937-949.

12. Wilson RM, Runciman WB, Gibberd RW, Harrison BT, Newby L, Hamilton JD. The Quality in Australian Health Care Study. Med J Aust. 1995;163(9):458-471.

13. Pucher PH, Aggarwal R, Darzi A. Surgical ward round quality and impact on variable patient outcomes. Ann Surg. 2014;259(2):222-226.

14. Fernando KJ, Siriwardena AK. Standards of documentation of the surgeon-patient consultation in current surgical practice. Br J Surg. 2001;88(2):309-312.

15. Neale G, Woloshynowych M, Vincent C. Exploring the causes of adverse events in NHS hospital practice. J R Soc Med. 2001;94(7):322-330.

16. Pucher PH, Aggarwal R. Re: Does Surgical Ward Round Quality Really Impact on Patient Outcomes? AnnSurg. 2016;263(1):e10.

17. Royal College of Physicians RCoN. Modern ward rounds: Good practice for multidisciplinary inpatient review. RCP, London; 2021.

18. O'Hare JA. Anatomy of the ward round. Eur J Intern Med. 2008;19(5):309-313.

19. Klingensmith ME. Ward rounds and patient outcome: be attentive or suffer the peril. Ann Surg. 2014;259(2):227-228.

20. Ghaferi AA, Birkmeyer JD, Dimick JB. Hospital volume and failure to rescue with high-risk surgery. Med care. 2011;49(12):1076-1081.

21. Page MJ, McKenzie JE, Bossuyt PM, Boutron I, Hoffmann TC, Mulrow CD, et al. The PRISMA 2020 statement: an updated guideline for reporting systematic reviews. BMJ. 2021;372:n71.

22. Moher D, Liberati A, Tetzlaff J, Altman DG. Preferred reporting items for systematic reviews and meta-analyses: the PRISMA statement. Ann Intern Med. 2009;151(4):264-269, w64.

23. Kellermeyer L, Harnke B, Knight S. Covidence and rayyan. J Med Libr Assoc. 2018;106(4):580.

24. Luchini C, Stubbs B, Solmi M, Veronese N. Assessing the quality of studies in meta-analyses: Advantages and limitations of the Newcastle Ottawa Scale. World J Meta-Anal. 2017;5(4):80-84.

25. Minozzi S, Cinquini M, Gianola S, Gonzalez-Lorenzo M, Banzi R. The revised Cochrane risk of bias tool for randomized trials (RoB 2) showed low interrater reliability and challenges in its application. J Clinepidemiolo. 2020;126:37-44.

26. Abraham J, Jaros J, Ihianle I, Kochendorfer K, Kannampallil T. Impact of EHR-based rounding tools on interactive communication: A prospective observational study. Int J Med Inform. 2019;129:423-429.

27. Acal Jimenez R, Swartz M, McCorkle R. Improving Quality Through Nursing Participation at Bedside Rounds in a Pediatric Acute Care Unit: A Pilot Project. JPediatrNurs. 2018;43:45-55.

28. Banfield DA, Adamson C, Tomsett A, Povey J, Fordham T, Richards SK. 'Take Ten' improving the surgical post-take ward round: a quality improvement project. BMJ Open Qual. 2018;7(1):e000045.

29. Alazzawi S, Silk Z, Saha UU, Auplish S, Masterson S. A ward round proforma improves documentation and communication. Br J Hosp Med (London, England : 2005). 2016;77(12):712-716.

30. Al-Mahrouqi H, Oumer R, Tapper R, Roberts R. Post-acute surgical ward round proforma improves documentation. BMJ Qual Improv Rep. 2013;2(1).

31. Armstrong EJ, Carpenter KJ. A Standardized Ward Round Proforma Improves Documentation in a Specialist Stroke Unit. Cureus. 2022;14(11):e31931.

32. Blucher KM, Dal Pra SE, Hogan J, Wysocki AP. Ward safety checklist in the acute surgical unit. ANZ J Surg. 2014;84(10):745-747.

33. Boland X. Implementation of a ward round pro-forma to improve adherence to best practice guidelines. BMJ Qual Improv Rep. 2015;4(1).

34. Brown N, Horne J, Low A. Improving documentation and junior doctor confidence on COVID-19 ward rounds using a ward round pro forma. Clin Med (Lond). 2021;21(Suppl 2):17-18.

35. Brown OS, Toi TH, Barbosa PR, Pookarnjanamorakot P, Trompete A. A patient-centred check sheet improves communication on the trauma ward round. Br JHospMed (Lond). 2019;80(8):472-475.

36. Byrd AS, McMahon PM, Vath RJ, Bolton M, Roy M. Integration of Mobile Devices to Facilitate Patient Care and Teaching During Family-Centered Rounds. Hosp Pediatr. 2018;8(1):44-48.

37. Cao V, Horn F, Laren T, Scott L, Giri P, Hidalgo D, et al. Patient-centered structured interdisciplinary bedside rounds in the medical ICU. Crit Care Med. 2016;44(12 Supplement 1):346.

38. Chow MY, Nikolic S, Shetty A, Lai K. Structured Interdisciplinary Bedside Rounds in an Australian tertiary hospital emergency department: Patient satisfaction and staff perspectives. Emerg Med Australas : EMA. 2019;31(3):347-354.

39. Christensen K, Janssens S, Beckmann M. Evaluation of a standardized ward round in a prenatal inpatient setting. Int J Gynaecol Obstet. 2017;136(3):357-361.

40. Christianson K, Kalinowski A, Bauer S, Liu Y, Titus L, Havas M, et al. Using Quality Improvement Methodology to Increase Communication of Discharge Criteria on Rounds. Hosp Pediatr. 2022;12(2):156-164.

41. Cifra CL, Houston M, Otto A, Kamath SS. Prompting Rounding Teams to Address a Daily Best Practice Checklist in a Pediatric Intensive Care Unit. Jt Comm J Qual Patietn Saf. 2019;45(8):543-551.

42. Clark NA, Burrus S, Richardson T, Sterner S, Queen MA. Implementation of a General Pediatric Clinical Rounding Checklist. HospPediatr. 2019;9(4):291-299.

43. Conroy KM, Elliott D, Burrell AR. Testing the implementation of an electronic process-of-care checklist for use during morning medical rounds in a tertiary intensive care unit: a prospective before-after study. AnnIntensive Care. 2015;5(1):60.

44. Crowson MG, Kahmke R, Ryan M, Scher R. Utility of Daily Mobile Tablet Use for Residents on an Otolaryngology Head & Neck Surgery Inpatient Service. J Med Sys. 2016;40(3):55.

45. De Bie AJR, Mestrom E, Compagner W, Nan S, van Genugten L, Dellimore K, et al. Intelligent checklists improve checklist compliance in the intensive care unit: a prospective before-and-after mixed-method study. Br JAnaesth. 2021;126(2):404-414.

46. Dhillon P, Murphy RKJ, Ali H, Burukan Z, Corrigan MA, Sheikh A, et al. Development of an adhesive surgical ward round checklist; a technique to improve patient safety. Ir Med J. 2011;104(10):1-3.

47. Dodek PM, Norena M, Wong H, Keenan S, Martin C. Assessing the Influence of Intensive Care Unit Organizational Factors on Outcomes in Canada: Is There Residual Confounding? J Intensive Care Ced. 2015;30(7):413-419.

48. Dolan R, Broadbent P. A quality improvement project using a problem based post take ward round proforma based on the SOAP acronym to improve documentation in acute surgical receiving. Ann Med Surg. 2016;5:45-48.

49. Duxbury O, Hili S, Afolayan J. Using a proforma to improve standards of documentation of an orthopaedic post-take ward round. BMJ Qual Improv Rep. 2013;2(1).

50. Eden EL, Rothenberger S, DeKosky A, Donovan AK. The Safe Discharge Checklist: A Standardized Discharge Planning Curriculum for Medicine Trainees. South Med J. 2022;115(1):18-21.

51. Efune PN, Morse RB, Sheehan M, Malone LM, Robertson TS, Darnell C. Improving Reliability to a Care Goal Rounding Template in the Pediatric Intensive Care Unit. Pediatr Qual Saf. 2018;3(6):e117.

52. Escamilla-Ocanas CE, Torrealba-Acosta G, Mandava P, Qasim MS, Gutierrez-Flores B, Bershad E, et al. Implementation of systematic safety checklists in a neurocritical care unit: a quality improvement study. BMJ Open Qual. 2022;11(4).

53. Feinman M, Hsu ATW, Taylor S, Torbeck L. Cutting the fat: Utilizing LEAN methodology to improve rounding efficiency of surgical residents. Am J Surg. 2022;223(6):1100-1104.

54. Fleischmann R, Duhm J, Hupperts H, Brandt SA. Tablet computers with mobile electronic medical records enhance clinical routine and promote bedside time: a controlled prospective crossover study. J Neurol. 2015;262(3):532-540.

55. Galloway GK, Choudhury SN. New take on the post-take ward round: a quality improvement project undertaken in a district general hospital. BMJ Open Qual. 2022;11(4).

56. Gilliland N, Catherwood N, Chen S, Browne P, Wilson J, Burden H. Ward round template: enhancing patient safety on ward rounds. BMJ Open Qual. 2018;7(2):e000170.

57. Glick AF, Foster LZ, Goonan M, Hart LH, Alam S, Rosenberg RE. Using Quality Improvement Science to Promote Reliable Communication During Family-Centered Rounds. Pediatr. 2022;149(4).

58. Hale G, McNab D. Developing a ward round checklist to improve patient safety. BMJ Qual Improv Rep. 2015;4(1).

59. Harman SM, Blankenburg R, Satterfield JM, Monash B, Rennke S, Yuan P, et al. Promoting Shared Decision-Making Behaviors During Inpatient Rounds: A Multimodal Educational Intervention. Acad Med.. 2019;94(7):1010-1018.

60. Johnston J, Stephenson J, Rajgopal A, Bhasin N. 'Every patient, every day': a daily ward round tool to improve patient safety and experience. BMJ Open Qual. 2022;11(3).

61. Justice LB, Cooper DS, Henderson C, Brown J, Simon K, Clark L, et al. Improving Communication During Cardiac ICU Multidisciplinary Rounds Through Visual Display of Patient Daily Goals. Pediatr Crit Care Med. 2016;17(7):677-683.

62. Kamara A, Henderson S, Rodrigo C, Dulay J. Does a Post-take Ward Round Proforma Lead to Sustainable Improvements in Quality of Documentation for Patients Admitted to the Medical Assessment Unit? Acute Med. 2006;5(3):108-111.

63. Kashyap R, Murthy S, Arteaga GM, Dong Y, Cooper L, Kovacevic T, et al. Effectiveness of a Daily Rounding Checklist on Processes of Care and Outcomes in Diverse Pediatric Intensive Care Units across the World. J Trop Pediatr. 2021;67(3):fmaa058.

64. Keller C, Arsenault S, Lamothe M, Bostan SR, O'Donnell R, Harbison J, et al. Patient safety ward round checklist via an electronic app: implications for harm prevention. Ir J Med Sci. 2018;187(3):553-559.

65. Khan A, Spector ND, Baird JD, Ashland M, Starmer AJ, Rosenbluth G, et al. Patient safety after implementation of a coproduced family centered communication programme: multicenter before and after intervention study. BMJ2018;363:k4764.

66. Koumoullis HD, Shapev M, Wong G, Gerring S, Patrinios G, Depasquale I, et al. Improving the quality of the daily ward round in a Plastic Surgery unit by adapting the SAFE Ward Round Tool of the Royal College of Surgeons of Edinburgh. Journal of Patient Safety and Risk Management. 2020;25(6):233-238.

67. Lepee C, Klaber RE, Benn J, Fletcher PJ, Cortoos P-J, Jacklin A, et al. The use of a consultant-led ward round checklist to improve paediatric prescribing: an interrupted time series study. Eur J Pediatr. 2012;171(8):1239-1245.

68. Licata J, Aneja R, Pasek T, Kyper C, Miller E, Spencer T, et al. A foundation for patient safety: Phase i implementation of interdisciplinary bedside rounds in the pediatric intensive care unit. Criti Care Med. 2011;39(SUPPL. 12):172.

69. Ludley A, Bahk A, Al-Shihabi A. A Structured Rounding Proforma in the Hyper Acute Stroke Unit (HASU): A Quality Improvement Project. J Health Qual2023;45(1):10-18.

70. Murphy D, Crowley R, Spencer A, Birch M. When can I go home? A prospective case control study to improve communication with patients regarding their diagnosis, treatment plan and likely discharge date. N Z Med J. 2015;128(1412):53-58.

71. Nassikas NJ, Monteiro JFG, Pashnik B, Lynch J, Carino G, Levinson AT. Intensive care unit rounding checklists to reduce catheter-associated urinary tract infections. Infect Control Hosp Epidemiol. 2020;41(6):680-683.

72. Newnham AL, Hine C, Rogers C, Agwu JC. Improving the quality of documentation of paediatric post-take ward rounds: the impact of an acrostic. Postgrad Med J. 2015;91(1071):22-5.

73. Ng J, Abdelhadi A, Waterland P, Swallow J, Nicol D, Pandey S, et al. Do ward round stickers improve surgical ward round? A quality improvement project in a high-volume general surgery department. BMJ Open Qual. 2018;7(3):e000341.

74. Palmer E, Richardson E, Newcombe H, Borg C-M. The F.R.I.D.A.Y.S. checklist - Preparing our patients for a safe weekend. BMJ Qual Improv Rep. 2014;2(2).

75. Parwaiz H, Trew CA, Whitham R, Aliaga-Crespo B, Mitra A, Harding I. Improving the weekend spinal ward round at a major trauma centre. Br J Hosp Med (Lond). 2022;83(6):1-5.

76. Pitcher M, Lin JT, Thompson G, Tayaran A, Chan S. Implementation and evaluation of a checklist to improve patient care on surgical ward rounds. ANZ J Surg. 2016;86(5):356-360.

77. Radhakrishnan NS, Lukose K, Cartwright R, Sleiman A, Matey N, Lim D, et al. Prospective application of the interdisciplinary bedside rounding checklist 'TEMP' is associated with reduced infections and length of hospital stay. BMJ Open Qual. 2022;11(4).

78. Redley B, Campbell D, Stockman K, Barnes S. A mixed methods quality evaluation of structured interprofessional medical ward rounds. Internal Med J. 2019.

79. Rehder KJ, Uhl TL, Meliones JN, Turner DA, Smith PB, Mistry KP. Targeted interventions improve shared agreement of daily goals in the pediatric intensive care unit. Pediatr Crit Care Med. 2012;13(1):6-10.

80. Sharma S, Peters MJ, Brierley J, Petros A, Pierce C, Skellett S, et al. 'Safety by DEFAULT': Introduction and impact of a paediatric ward round checklist. Crit Care. 2013;17(5):R232.

81. Shirreff L, Husslein H, Lefebvre GG, Shore EM. Introduction of Physician-Nurse Bedside Rounding and Ward Task List to Improve Quality of Care in Gynaecology: Prospective, Single-Blinded, Pre- and Post-Intervention Study. J Obstet Gynaecol Can. 2019;41(8):1108-14.

82. Simon K, Sankara IR, Gioe C, Newcomb P. Including Family Members in Rounds to Improve Communication in Intensive Care. J Nurs Care Qual. 2021;36(1):25-31.

83. Southwick F, Lewis M, Treloar D, Cherabuddi K, Radhakrishnan N, Leverence R, et al. Applying athletic principles to medical rounds to improve teaching and patient care. Acad Med. 2014;89(7):1018-1023.

84. Spaner D, Caraiscos VB, Muystra C, Furman ML, Zaltz-Dubin J, Wharton M, et al. Use of Standardized Assessment Tools to Improve the Effectiveness of Palliative Care Rounds: A Quality Improvement Initiative. J Palliat Care. 2017;32(3-4):134-140.

85. Stroud MH, Moss MM, Gilliam CH, Honeycutt M, Frost M, Green JW. Introduction of a rounding sticker improves care and reduces infection rates in the Pediatric Intensive Care Unit (PICU). J Ark Med Soc. 2012;109(6):114-117.

86. Sunkara P, Islam T, Bose A, Rosenthal GE, Chevli P, Jogu H, Tk LA, Huang CC, Chaudhary D, Beekman D, Dutta A, Menon S, Speiser JL. Impact of structured interdisciplinary bedside rounding on patient outcomes at a large academic health centre. BMJ Qual Saf. 2020 Jul;29(7):569-575

87. Talia AJ, Drummond J, Muirhead C, Tran P. Using a Structured Checklist to Improve the Orthopedic Ward Round: A Prospective Cohort Study. Orthopedics. 2017;40(4):e663-e667.

88. Thompson AG, Jacob K, Fulton J, McGavin CR. Do post-take ward round proformas improve communication and influence quality of patient care? Postgraduate Med J. 2004;80(949):675-676.

89. Torregrosa L, Ariza A, Villarreal L, Cabrera Vargas LF. Development and application of an inpatient traffic lights classification to improve the surgical ward round quality. Am J Surg. 2022;223(5):1010-2.

90. Trahan C, Hui AY, Binepal N. Standardization of rounds on a general paediatric ward: Implementation of a checklist to improve efficiency, quality of rounds, and family satisfaction. PaediatriChild Health. 2022;27(2):111-117.

91. Tranter-Entwistle I, Best K, Ianev R, Beresford T, Laws P, McCombie A. Introduction and validation of a surgical ward round checklist to improve surgical ward round performance in a tertiary vascular service. ANZ J Surg. 2020;90(7-8):1358-1363.

92. Urisman T, Garcia A, Harris HW. Impact of surgical intensive care unit interdisciplinary rounds on interprofessional collaboration and quality of care: Mixed qualitative-quantitative study. Intensive Crit Care Nurs. 2018;44:18-23.

93. Vukanic D, Kelly EG, Cleary SM. Does an Orthopedic Ward Round Pro Forma Improve Inpatient Documentation? J Patient Saf. 2020.

94. Weiss CH, Moazed F, McEvoy CA, Singer BD, Szleifer I, Amaral LA, Kwasny M, Watts CM, Persell SD, Baker DW, Sznajder JI, Wunderink RG. Prompting physicians to address a daily checklist and process of care and clinical outcomes: a single-site study. Am J Respir Crit Care Med. 2011 Sep 15;184(6):680-686.

95. Wright DN. Does a post-take ward round proforma have a positive effect on completeness of documentation and efficiency of information management? Health Informatics J. 2009;15(2):86-94.

96. Xu A, Chan LY, Abedin M, Sivapathasuntharam D. Use of a proforma to improve documentation of the post-take ward round and encourage initiation of the comprehensive geriatric assessment in the care of the older people's service. Br J Hosp Med (Lond). 2021;82(1):1-6.

97. Becker C, Gamp M, Schuetz P, Beck K, Vincent A, Hochstrasser S, et al. Effect of Bedside Compared With Outside the Room Patient Case Presentation on Patients' Knowledge About Their Medical Care : A Randomized, Controlled, Multicenter Trial. Ann Intern Med. 2021. 174(9):1282-1292.

98. Cavalcanti AB, Bozza FA, Machado FR, Salluh JIF, Campagnucci VP, Vendramim P, et al. Effect of a quality improvement intervention with daily round checklists, goal setting, and clinician prompting on mortality of critically ill patients: A randomized clinical trial. JAMA. 2016;315(14):1480-1490.

99. Clarke-Pounder JP, Boss RD, Roter DL, Hutton N, Larson S, Donohue PK. Communication intervention in the neonatal intensive care unit: can it backfire? J Palliat Med.2015;18(2):157-161.

100. Cox ED, Jacobsohn GC, Rajamanickam VP, Carayon P, Kelly MM, Wetterneck TB, et al. A Family-Centered Rounds Checklist, Family Engagement, and Patient Safety: A Randomized Trial. Pediatr. 2017;139(5).

101. Donovan AK, Spagnoletti C, Rothenberger S, Corbelli J. The impact of residents sitting at the bedside on patient satisfaction during team rounds. Patient Educ Couns. 2020;103(6):1252-1254.

102. Ellison LM, Nguyen M, Fabrizio MD, Soh A, Permpongkosol S, Kavoussi LR. Postoperative robotic telerounding: a multicenter randomized assessment of patient outcomes and satisfaction. Arch Surg. 2007;142(12):1177-1181.

103. Finn KM, Metlay JP, Chang Y, Nagarur A, Yang S, Landrigan CP, et al. Effect of Increased Inpatient Attending Physician Supervision on Medical Errors, Patient Safety, and Resident Education: A Randomized Clinical Trial. JAMA Intern Med. 2018;178(7):952-959.

104. Jaberi AA, Zamani F, Nadimi AE, Bonabi TN. Effect of family presence during teaching rounds on patient's anxiety and satisfaction in cardiac intensive care unit: A double-blind randomized controlled trial. J Educ Health Promot. 2020;9:22.

105. Lienard A, Merckaert I, Libert Y, Bragard I, Delvaux N, Etienne A-M, et al. Transfer of communication skills to the workplace during clinical rounds: impact of a program for residents. PloS one. 2010;5(8):e12426.

106. Osborn R, Grossman M, Berkwitt A. The Effect of Sitting Versus Standing on Family Perceptions of Family-Centered Rounds. Hosp Pediatr. 2021;11(11):e313-e316.

107. Roberts RP, Blackwell SC, Brown KM, Pedroza C, Sibai BM, Tyson JE. Early Compared With Delayed Physician Rounds on Patient Satisfaction of Postpartum Women: A Randomized Controlled Trial. Obstet Gynecol. 2016;128(2):381-386.

108. Read J, Perry W, Rossaak JI. Ward round checklist improves patient perception of care. ANZ J Surg. 2021;91(5):854-859.

109. Eden E, Rothenberger SD, DeKosky A, Donovan AK. The safe discharge curriculum: How we standardized residents' approach to hospital discharge. Journal of General Internal Medicine. 2018;33(2 Supplement 1):372.

110. McKenna HP, Ashton S, Keeney S. Barriers to evidence‐based practice in primary care. J Adv Nurs. 2004;45(2):178-189.

111. Mohan DR, Kumar KS. A study on the satisfaction of patients with reference to hospital services. Int J Bus Econ Manag Res. 2011;1(3):15-25.

112. Schoenfelder T, Klewer J, Kugler J. Determinants of patient satisfaction: a study among 39 hospitals in an in-patient setting in Germany. Int J Qual Health Care. 2011;23(5):503-509.

113. Manias E, Kusljic S, Wu A. Interventions to reduce medication errors in adult medical and surgical settings: a systematic review. Ther Adv Drug Saf. 2020;11:2042098620968309.

114. Donaldson MS, Corrigan JM, Kohn LT. To err is human: building a safer health system. 2000.

115. UK NGC. Structured ward rounds. Emergency and acute medical care in over 16s: service delivery and organisation: National Institute for Health and Care Excellence (NICE); 2018.

116. ES S. Applying human factors methods to clinical risk management in obstetrics. Br J Obstet. 1997;104:1225-1232.

117. Vincent C, Taylor-Adams S, Chapman EJ, Hewett D, Prior S, Strange P, et al. How to investigate and analyse clinical incidents: clinical risk unit and association of litigation and risk management protocol. BMJ. 2000;320(7237):777-781.

118. Vaucher C, Bovet E, Bengough T, Pidoux V, Grossen M, Panese F, et al. Meeting physicians' needs: a bottom-up approach for improving the implementation of medical knowledge into practice. Health Res Policy Syst. 2016;14(1):49.

119. Menon D, Stafinski T, editors. Bridging the “know-do” gap in healthcare priority-setting: what role has academic research played? Healthcare Management Forum; 2005: SAGE Publications Sage CA: Los Angeles, CA.
